# Neural Circuit Interactions between the Dorsal Raphe Nucleus and the Lateral Hypothalamus: An Experimental and Computational Study

**DOI:** 10.1371/journal.pone.0088003

**Published:** 2014-02-06

**Authors:** Jaishree Jalewa, Alok Joshi, T. Martin McGinnity, Girijesh Prasad, KongFatt Wong-Lin, Christian Hölscher

**Affiliations:** 1 School of Biomedical Sciences, University of Ulster, Coleraine, Northern Ireland, United Kingdom; 2 Intelligent Systems Research Centre, University of Ulster, Magee Campus, Londonderry, Northern Ireland, United Kingdom; 3 Division of Biomedical and Life Sciences, Faculty of Health and Medicine, Lancaster University, Lancaster, United Kingdom; McGill University, Canada

## Abstract

Orexinergic/hypocretinergic (Ox) neurotransmission plays an important role in regulating sleep, as well as in anxiety and depression, for which the serotonergic (5-HT) system is also involved in. However, little is known regarding the direct and indirect interactions between 5-HT in the dorsal raphe nucleus (DRN) and Ox neurons in the lateral hypothalamus (LHA). In this study, we report the additional presence of 5-HT_1B_R, 5-HT_2A_R, 5-HT_2C_R and fast ligand-gated 5-HT_3A_R subtypes on the Ox neurons of transgenic Ox-enhanced green fluorescent protein (Ox-EGFP) and wild type C57Bl/6 mice using single and double immunofluorescence (IF) staining, respectively, and quantify the colocalization for each 5-HT receptor subtype. We further reveal the presence of 5-HT_3A_R and 5-HT_1A_R on GABAergic neurons in LHA. We also identify NMDAR1, OX_1_R and OX_2_R on Ox neurons, but none on adjacent GABAergic neurons. This suggests a one-way relationship between LHA’s GABAergic and Ox neurons, wherein GABAergic neurons exerts an inhibitory effect on Ox neurons under partial DRN’s 5-HT control. We also show that Ox axonal projections receive glutamatergic (PSD-95 immunopositive) and GABAergic (Gephyrin immunopositive) inputs in the DRN. We consider these and other available findings into our computational model to explore possible effects of neural circuit connection types and timescales on the DRN-LHA system’s dynamics. We find that if the connections from 5-HT to LHA’s GABAergic neurons are weakly excitatory or inhibitory, the network exhibits slow oscillations; not observed when the connection is strongly excitatory. Furthermore, if Ox directly excites 5-HT neurons at a fast timescale, phasic Ox activation can lead to an increase in 5-HT activity; no significant effect with slower timescale. Overall, our experimental and computational approaches provide insights towards a more complete understanding of the complex relationship between 5-HT in the DRN and Ox in the LHA.

## Introduction

Mood and neuropsychiatric disorders such as depression have a close relationship with sleep disturbances, and are instantiated by the overlap of emotional processing and the sleep-wake regulation neuronal circuitries [1]. The neuropeptide hormone orexin/hypocretin (Ox) has been known to regulate sleep and its deregulation is related to narcolepsy, and novel drugs to facilitate sleep induction by activating Ox receptors are currently under development [2], [3], [4]. Recent studies also suggest a role for Ox in depression, emotional processing, reward seeking behaviour and in the regulation of endocrine functions [5], [6], [7], [8], [9], [10]. Ox neurons comprising of neuropeptides Ox A and Ox B are found predominantly in the lateral hypothalamus (LHA) [11], [12] and are known to function through OX_1_R and OX_2_R G-protein coupled receptors, respectively [13], [14], [15], [16].

The neurotransmitter/neuromodulator 5-hydroxytryptamine (5-HT) released by serotonergic neurons, substantially located in the midbrain’s dorsal raphe nucleus (DRN) is often associated with mood and emotional processing, and its dysfunction is related to mood and neuropsychiatric disorders [17], [18], [19]. In addition, perturbations of 5-HT have also been found to influence sleep [18], [20], [21], [22]. Numerous drugs to treat depression are already on the market that target 5-HT neurotransmission [23], [24].

It is important to understand how drugs that target Ox and/or 5-HT systems alter neuronal activity and signal transmission in order to understand the underlying mechanisms of antidepressive and sleep inducing effects. Therefore, we set out to map what subtypes of 5-HT receptors are expressed by Ox neurons, and how neuronal transmission and signal transduction in neuronal circuits may be controlled by these receptors.

Up till now, only the (inhibitory) 5-HT_1A_R has so far been found in LHA’s Ox neurons [25], [26], [27]. In addition to their inhibitory (5-HT_1A_) autoreceptors [28], 5-HT also excites the GABAergic inhibitory neurons within the DRN for self-regulation [29], [30], [31], [32], [33]. Within the LHA, in addition to their self-excitatory Ox autoreceptors [4], Ox neurons can send direct long-range excitation to 5-HT neurons, and the GABAergic neurons in the DRN [31], mediated by both OX_2_R and OX_1_R [30], [32]. Thus, Ox can have both direct and indirect influences on the DRN’s 5-HT neurons. However, it remains unknown whether 5-HT can reciprocally indirectly influence LHA’s Ox neurons by influencing the LHA’s GABAergic neurons, and whether this connection is effectively excitatory or inhibitory. It is also not known whether Ox can innervate its local GABAergic neurons similar to how 5-HT neurons excite their local GABAergic neurons [34].

Knowledge of direct and indirect circuit connections is important to provide a more complete understanding of diversified neural circuit dynamics and regulations within the DRN-LHA system [35], [36]. Furthermore, it is not known how the interplay between fast synaptic transmission and slow currents induced by 5-HT and Ox can affect the relay of information in these circuits. In this work, primarily through immunofluorescence (IF) staining, we attempt to map out a more complete neural circuit between the principal (5-HT and Ox) and non-principal (GABAergic and glutamatergic) neurons. Based on some of these findings, we present a computational model to investigate possible neural circuit dynamics between the DRN and LHA. As our goal in the modelling is to understand how the various neural circuit architecture and connectivity timescale affect the DRN-LHA activity, we keep the model as simple as possible. Hence we make use of population-based or “mean-field” firing-rate type approach, which compromises between previous biophysical models [37], [38], [39], [40] and more abstract mathematical models [41], [42], [43].

Our experimental results reveal various other 5-HT receptor subtypes expressed in Ox and GABAergic neurons in the LHA, provide more evidence to support a unidirectional relationship between these LHA’s neurons, and suggest that Ox can project to DRN’s 5-HT neurons indirectly through local non-5-HT neurons. Our computational modelling results show that if 5-HT is weakly excitatory or inhibitory on LHA’s GABAergic neurons, the network can exhibit slow oscillation. This is not observed if the connection is strongly excitatory. Furthermore, we show the importance of the timescale for the Ox-to-DRN connection during transient behaviour.

## Materials and Methods

### Animals

Twelve-week-old C57BL/6 male mice (n = 4) were used for each qualitative experiment and n = 6 mice for each quantification experiment described in this study. An orexin/enhanced green fluorescent protein (Ox-EGFP) breeder pair was a kind gift from Prof. Takeshi Sakurai (Kanazawa University, Japan). Brain sections from these mice show green fluorescence in the Ox neurons when excited at 488 nm wavelength. Breeding was set up in-house and the male pups were aged to ten-twelve weeks before the start of the experiments. Animals were maintained on a 12/12 h light/dark cycle (lights on at 8∶00 A.M., off at 8∶00 P.M.), in a temperature-controlled room (21.5±1°C). Animals received food and water *ad libitum*. All animal experiments were licenced by the UK Home Office in accordance with the Animals (Scientific Procedures) Act of 1986 and in agreement with UK and EU laws.

### Perfusion, Fixation and Sectioning

Mice were anaesthetised with pentobarbitone (0.3 ml; Euthanal, Bayer AG, Leverkusen, Germany) and perfused transcardially with 0.1 M PBS (pH 7.4) buffer followed by ice-cold 4% paraformaldehyde in PBS. The brains were removed and fixed in 4% paraformaldehyde for at least 24 hr and cryoprotected in a 30% sucrose solution in PBS overnight at 4°C. Brains were then snap frozen using Envirofreez, and coronal sections of 45 µm thickness were cut using a Leica cryostat. According to the mouse brain atlas by Paxinos and Franklin (2004), LHA and DRN sections were cut at a depth of −0.34 mm to −2.80 mm bregma and −4.04 mm to −5.20 mm bregma respectively. Sections were chosen according to the stereological rules, with the first section taken at random and every sixth section afterward. In the case of LHA, by taking every 6^th^ section (in total 54 sections per LHA per brain half), at least 10–11 sections were taken per immunostaining experiment (n = 4). In the case of DRN, by taking every 3^rd^ section (in total 25 sections per DRN per full brain), at least 11–12 sections were taken per immunostaining experiment (n = 4). 10–11 sections from 4 mice brain halves (in case of LHA) and full brain (in case of DRN) were processed so, at least 40 sections were considered in total for every immunostaining experiment.

### Immunohistochemistry

Single, double or triple immunofluorescence (IF) staining experiments were performed on 45 µm free-floating sections using primary antibodies: i) affinity purified goat polyclonal Ox-A (C-19) IgG (1∶400 dilution, Santa Cruz, sc-8070), raised against a peptide mapping at the C-terminus of Orexin-A of human origin; ii) rabbit polyclonal anti-5HT_1B_ receptor IgG (1∶500 dilution, Abcam, ab102700) raised against a synthetic peptide taken from within the region 230–280 (designed to the 3rd cytoplasmic domain) of the human 5HT_1B_ receptor conjugated to an immunogenic carrier protein; iii) rabbit polyclonal anti-5HT_1A_ receptor IgG (1∶500 dilution, Abcam, ab79230) raised against a synthetic peptide from the 3rd cytoplasmic domain of mouse 5HT_1A_ receptor, conjugated to an immunogenic carrier protein [44]; iv) rabbit polyclonal anti-5HT_2A_ receptor IgG (1∶500 dilution, Abcam, ab16028) raised against a synthetic peptide conjugated to KLH derived from within residues 1–100 of rat 5HT_2A_ receptor [45], [46], [47], [48], [49]; v) rabbit polyclonal anti-5HT_2C_ receptor IgG (1∶500 dilution, Abcam, ab32172) raised against a synthetic peptide derived from within residues 400 to the C-terminus of rat 5HT2C receptor [48], [50], [51], [52]; vi) rabbit polyclonal anti-HTR3A receptor IgG (1∶500 dilution, Sigma-Aldrich, AV13046) raised against a synthetic peptide corresponding to a region of human HTR3A with an internal ID of P01375. The immunogen for anti-HTR3A antibody was a synthetic peptide directed towards the N-terminal of human HTR3A, with the following sequence: LLWVQQALLALLLPTLLAQGEARRSRNTTRPALLRLSDYLLTNYRKVGRP; vii) rabbit monoclonal anti-NMDAR, clone 1.17.2.6 IgG (1∶500 dilution, Millipore, AB9864R) raised against linear peptide corresponding to human NMDAR1; viii) guinea pig polyclonal anti-GABA IgG (1∶400 dilution, Millipore, AB175) raised against GABA coupled to KLH via glutaraldehyde; ix) rabbit polyclonal anti-Orexin receptor 1 IgG (1∶500 dilution, Abcam, ab83960) raised against a synthetic peptide from the internal region (250–300) of mouse Orexin receptor 1 conjugated to an immunogenic carrier; x) rabbit polyclonal anti-Orexin receptor 2 IgG (1∶500 dilution, Abcam, ab85899) raised against a synthetic peptide from the C terminal region (350–415) of human Orexin receptor 2 conjugated to an immunogenic carrier; xi) rabbit monoclonal PSD-95 (D27E11) XP IgG (1∶500 dilution, Cell Signaling Technology, 3450S) raised against a synthetic peptide corresponding to residues surrounding Gly99 of human PSD95; xii) rabbit polyclonal Anti-Gephyrin IgG (1∶500 dilution, Abcam, ab32206) raised against a synthetic peptide conjugated to KLH derived from within residues 700 to the C-terminus of mouse Gephyrin.

All primary antibodies used in this study were previously characterized and their specificity was verified according to the respective manufacturer. After blocking in 1% BSA and 5% donkey normal serum in TBS buffer (pH 8, Sigma Aldrich) to avoid nonspecific antibody binding, sections were incubated in the primary antibody overnight at 4°C. The following day, sections were incubated in the secondary antibody for an hour at room temperature and mounted using Vectashield mounting medium (Vector Laboratories) on the slides coated with 3-aminopropyl tri-ethoxy silane (Sigma Aldrich). For double IF stainings, a simultaneous method was used where sections were incubated with two primary antibodies together for 48 hrs at 4°C. In the case of triple IF stainings, three primary antibodies were added together and sections were incubated for 72 hrs at 4°C. Labeled donkey IgG (H+L) anti-goat Alexa Fluor 488 (1∶800 dilution, Cat. # A11055, Molecular Probes), anti-rabbit Alexa Fluor 546 (1∶1000 dilution, Cat. # A11056, Molecular Probes), anti-chicken CF 594 (1∶1000 dilution, Cat. # BTIU20167, Biotium) and anti-guinea pig CF 633 (1∶1000 dilution, Cat. # BTIU20171, Biotium) secondary antibodies were used in the study. Negative controls were performed for single IF staining by omitting the primary antibody and for double/triple IF staining by omitting the primary and secondary antibody.

### Antigen Retrieval

Antigen retrieval was done while performing NMDAR1 IF staining. Sections were incubated in 10 mM sodium citrate (pH 6) at 80°C for 30 min, before blocking the sections.

### Microscopy

#### Fluorescence microscopy

All single IF stainings in EGFP brain sections for 5-HT receptors on the Ox neurons in the LHA were visualized using Qimaging (Chromaphor). Microscopy was performed using an Olympus BX51 (Surveyor version 5.5.5.30, automated specimen scanning for the OASIS automation control system).

#### Confocal microscopy

Imaging was performed using a confocal microscope (Leica Microsystems; SP5 LAS IF Software). For quantification experiments, three sections of similar density of Ox neurons in the LHA were analyzed per brain (n = 6). 4–5 images were obtained from each section thus, 70–90 images were analyzed for each quantification experiment.

### Co-localization Quantification

For quantification of the 5HT receptor subtype on the Ox neurons in LHA, images were acquired using 63× objective in a Leica SP5 confocal microscope. Once the conditions such as photomultiplier gain for each channel and pinhole settings were adjusted to minimize background noise and saturated pixels, parameters were kept constant for all acquisitions. Triple-stained images were obtained by sequential scanning for each channel to eliminate the cross talk of chromophores and to ensure reliable quantification of co-localization. Ambiguity and inconsistency are the two major issues affecting colocalization analysis. In the context of digital imaging, colocalization means the colours emitted by the fluorescent molecules occupy the same pixel in the image [53], [54]. Therefore, we have used the JACoP (Just Another Colocalization Plug-in) tool of Image J for colocalization analysis. The degree of Ox neuron (Alexa488, green) signal colocalizing with 5HT receptor (Alexa546, red) signal was quantified on single-plane 8-bit color images using the JACoP plugin [55]. A simple way of measuring the dependency of pixels in dual-channel images is to plot the pixel grey values of two images against each other. The intensity of a given pixel in the green image is used as the x-coordinate of the scatter plot and the intensity of the corresponding pixel in the red image as the y-coordinate. Results in JACoP are displayed in a pixel distribution diagram called a scatter plot or fluorogram in addition to the calculated co-localization coefficients such as Pearson’s and Overlap coefficient.

Pearson’s correlation coefficient (Rr) is the most quantitative estimate of colocalization that depends on the amount of colocalized signals in both channels in a nonlinear manner and is a well-defined and commonly accepted means for describing the extent of overlap between image pairs [56]. It is used for describing the correlation of the intensity distributions between channels. It takes into consideration only similarity between shapes, while ignoring the intensities of signals. The values of Pearson’s coefficient range from −1 to 1, with values from 0.5 to 1.0 indicating colocalization and −1.0 to 0.5 indicating no-colocalization. As Pearson’s Correlation does some averaging of pixel information and can return negative values another method, the Overlap Coefficient, is simultaneously used to describe overlap. Manders’ overlap coefficient (R) is based on the Pearson’s correlation coefficient with average intensity values being taken out of the mathematical expression. This new coefficient varies from 0 to 1, with values from 0.6 to 1.0 indicating colocalization and 0 to 0.6 indicating no colocalization. Overlap coefficient according to Manders indicates an overlap of the signals and thus represents the true degree of Colocalization. Costes randomization (number of randomization rounds = 1000) was used to exclude any co-localization of pixels that might have occurred due to chance [57]. P-value for each image pair was 100.0% (calculated from the fitted data).

### Statistics

Statistical analyses were performed using Prism 5 (GraphPad software Inc. USA) with the level of probability set at 95% and the results are expressed as means±SEM. Data for 5-HT receptor quantification was analysed by two-tailed unpaired t-test.

### Computational Model

To investigate the consequences of the DRN-LHA circuit architecture on systems dynamics, we implement neural network model that is an extension and modification of our previous model [58]. The aim of the model is to understand how the circuit connectivity and timescale affect the DRN-LHA activity.

#### Neural units

Our neural network model consists of 4 populations of neurons, namely, Ox neurons in the LHA, local LHA inhibitory GABAergic neurons, 5-HT neurons in the DRN, and local DRN inhibitory GABAergic neurons. Glutamatergic neurons will be ignored in this work primarily due to the evidence showing that glutamatergic effects in DRN are locally weaker when compared to local GABAergic influence [59], and that we can implicitly encompass the effects of the LHA’s glutamatergic neurons on Ox neurons [4] with self-excitatory Ox connections. Furthermore, incorporating two additional (glutamatergic) neural populations can lead to more free parameters in the model.

The chosen model is of the population-averaged or “mean-field” firing rate type model [60], [61], [62], [63]. This simplifies a population of neurons into its representative unit. The 4 neural populations to be considered are the LHA’s Ox and GABAergic neural populations, and the DRN’s 5-HT and GABAergic neural populations. With support from electrophysiological data, the input-output or current-frequency relationship (*f-I* curve) for each neural population can be described by threshold-linear functions [64], [65]:


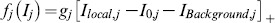
(1)

where *f_j_* and *(I_Local,j_ – I_Background,j_)* denote the population-averaged firing rate activity and total afferent input of the *j^th^* neural population, respectively. *I_Background,j_* is the background current, consisting of inputs from the rest of the brain areas. *g_j_* and *I_0,j_* determine the input-output slope and the current threshold, respectively. The threshold-linear function *[z]_+_ = z* if *z>0*, and *0* otherwise. Based on their known neuronal electrophysiological properties [4], [27], [66]), we determine and constrain the values of the *g_j_’s* (i.e. g_5-HT_ and g_GABA_ for DRN; g_GABA_ and g_Ox_ for LHA) and the *I_0,j_’s*, while consider *I_Background,j_* as a free parameter. Note that we have assumed the dynamics of the neural population (neuronal membrane time constant ∼ 10 ms) to be relatively instantaneous and slaved to the much slower timescale of the connections (∼ s) [63]. This simplification reduces 4 model parameters (neuronal membrane time constants) and 4 dynamical (differential) equations for the 4 neuronal types [60].

2.8.2. Inputs and connections: Since each neural unit receives inputs from self-feedback and from 2 other neural units (e.g. 5-HT neuronal population receives both afferent inputs from DRN’s GABAergic neurons and longer range connection from Ox neurons), the local afferent input (minus the background input) can be described by:
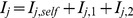
(2)


where the subscript “self” denotes a self-feedback connection (e.g. due to autoreceptors in Ox and 5-HT neural populations, or GABAergic synapses in the two inhibitory neural populations), and the subscript “1″ or “2″ denotes the afferent inputs due to the other 2 neural populations. For local GABAergic neurons, they receive self-inhibition, and projections from their local principal neural population (5-HT or Ox if from DRN or LHA, respectively), and long-range projection from the other brain region (LHA or DRN). If the net effect of the *i^th^* neural population on the *j^th^* neural population is inhibitory or excitatory, the coefficient in front of *I_j,i_* will be −*1* or *+1*, respectively. For example, suppose the 5-HT neural population receives inhibitory autoreceptor influence, direct projection from Ox neurons, and local GABAergic influence. Then,
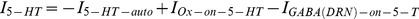
(3)


To simulate the phasic response of the circuit, an extra term *I_stim_* is added to the 5-HT or/and Ox inputs 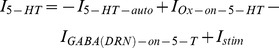
(4)


The dynamics of each input are filtered by its (synaptic/effective) time constant (*τ*_syn, j, i_) as follows: 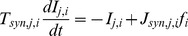
(5)


In principle, there are 10 such similar dynamical equations describing the currents caused by ionotropic (4 equations) and metabotropic (6 equations) receptors. The synaptic time constants associated with the ionotropic receptors are obtained from electrophysiological data while the effective metabotropic time constants are deduced from the associated G protein-coupled inwardly-rectifying potassium (GIRK) current, or if unavailable, the temporal change in firing rate due to the injection of 5-HT or Ox [67]. For simplicity, a simple linear relationship is assumed between the input current *I_j,i_* and activity level *f_i_*. The other important parameter, *J_syn,j,i_* is the connection strength within or between the neuronal populations. Under steady state condition (*dI_j,i_*/dt = 0), *J_syn,j,i_* is defined as the ratio of the current *I_j,i_* and the associated (presynaptic) activity *f_i_*. These currents are obtained from various experiments [4], [26], [27], [31], [68], [69]. The *in vivo* baseline firing rates (*f_i_*) for the Ox and GABAergic populations in LHA are ∼5 Hz (3–8 Hz in experiments) [70], [71], [72]. In DRN, the baseline neuronal firing rate of 5-HT and GABAergic neuronal population is ∼5 and ∼15 Hz, respectively [73]. The relationship among these baseline activities will be used to constrain our model parameters, namely the *J_i_*, *g_i_* and *I_j,0_* (Table 1). Consistent with our assumption on ignoring the relatively much faster neuronal membrane dynamics (∼10 s ms), we shall also ignore the dynamics for the relatively fast GABAergic synapses (∼4 ms), assuming they attain instantaneous steady states. This further reduces 4 dynamical equations in describing the associated currents.

**Table 1:** Time constants, currents of all the neuronal groups, values are deduced from experiments, and by using above constraints and Connection strength (C. strength), and background current of all the neuronal groups.

A. Time constants, currents of all the neuronal groups, values are deduced from experiments, and by using above constraints.			
Parameter	Description	Value	Reference, remarks
τ_5-HT-auto_	5-HT_1A_ autoreceptor time constant	1 s	[90], [91]
I_5-HT-auto_	5-HT_1A_ autoreceptor induced current amplitude	80 pA	[68]
I_GABA(DRN)-on-5-HT_	GABA_A_ mediated current amplitude in 5-HT neurons	70 pA	[31]
τ_Ox-on-5-HT_	Ox_1,2_ induced current time constant on 5-HT neurons	60 s	[31]
I_Ox-on-5-HT_	Ox_1,2_ induced current amplitude on 5-HT neurons	75 pA	[31]
I_GABA(DRN)-on-GABA(DRN)_	GABA_A_ mediated current amplitude in DRN’s GABAergic neurons	70 pA	I_GABA(DRN)-on-GABA(DRN)_ ∼ I_GABA(DRN)-on-5-HT_
τ_5-HT-on-GABA(DRN)_	5-HT induced current time constant in GABAergic neurons in DRN	60 s	[92]
I_5-HT-on-GABA(DRN)_	5-HT induced current amplitude in GABAergic neurons inDRN	50 pA	[92]
τ_Ox-on-GABA(DRN)_	Ox induced current timescale on GABAergic neurons in DRN	5 s	[31]
I_Ox-on-GABA(DRN)_	Ox induced current amplitude in GABAergic neurons in DRN	25 pA	[31]
τ_Ox-auto_	Ox_2_ autoreceptor time constant	10 s	[4]
I_Ox-auto_	Ox_2_ autoreceptor induced current amplitude	30 pA	[4]
I_GABA(LHA)-on-Ox_	GABAergic induced current amplitude in Ox neurons	590 pA	[93]
τ_5-HT-on-Ox_	5-HT_1A_ induced current time constant on Ox neurons	2 s	[26]
I_5-HT-on-Ox_	5-HT_1A_ induced current amplitude in LHA	32 pA	[26]
I_ GABA(LHA)-on-GABA(LHA)_	GABA mediated current amplitude in LHA’s GABAergic neurons	70 pA	I_GABA(LHA)-on-GABA(LHA)_ ∼ I_GABA(DRN)-on-Ox_
τ_Ox-on-GABA(LHA)_	Ox induced current time constant on LHA’s GABAergic neurons	10 s	τ_Ox-on-GABA(LHA)_ ∼ τ_Ox-auto_
I_Ox-on-GABA(LHA)_	Ox induced current amplitude in LHA’s GABAergic neurons	30 pA	I_Ox-on-GABA(LHA)_ ∼ I_Ox-auto_
τ_5-HT-on-GABA(LHA)_	5-HT induced current time constant in LHA’s GABAergic neurons	2 s	τ_5-HT-on-GABA(LHA)_ ∼τ_5-HT-on-Ox_
I_5-HT-on-GABA(LHA)_	5-HT induced current amplitude inLHA’s GABAergic neurons	32 pA	I_5-HT-on-GABA(LHA)_ ∼I_5-HT-on-Ox_
g_5-HT_	Slope of input-output function of 5-HT neurons	0.033 Hz/pA	[64]
g_Ox_	Slope of input-output function of LHA’s GABAergic neurons	0.205 Hz/pA	[65]
g_GABA(DRN)_	Slope of input-output function of DRN’s GABAergic neurons	0.061 Hz/pA	[64]
g_GABA(LHA)_	Slope of input-output function of Ox neurons	0.195 Hz/pA	[65]
I_5-HT,0_	Current threshold of 5-HT neurons	0.13 pA	[64]
I_Ox,0_	Current threshold of Ox neurons	0 pA	[65]
I_GABA(DRN),0_	Current threshold of DRN’s GABAergic neurons	0 pA	[65]
I_GABA(LHA),0_	Current threshold of LHA’s GABAergic neurons	0 pA	[64]
a	Stimulus current amplitude applied to the 5-HT or Oxneurons	150 pA	2-fold activity increase in the 5-HT neurons
–	Duration of the applied stimulus	0.5 s	Behavioural timescale
**B. Connection strength (C. strength), and background current of all the neuronal groups.**			
J_5-HT-auto_	C. strength of 5-HT_1A_ autoreceptors	16 pA/Hz	*
J_GABA(DRN)-to-5-HT_	C. strength of GABAergic on 5-HT neurons in DRN	5 pA/Hz	*
J_Ox-to-5-HT_	C. strength of Ox neurons on 5-HT neurons	15 pA/Hz	*
J_GABA(DRN)-to-GABA(DRN)_	C. strength of GABAergic neurons in DRN	5 pA/Hz	*
J_5-HT-to-GABA(DRN)_	C. strength of 5-HT on GABAergic neurons in DRN	10 pA/Hz	*
J_Ox-to-GABA(DRN)_	C. strength of Ox on GABAergic neurons in DRN	5 pA/Hz	*
J_Ox-auto_	C. strength of Ox_2_ autoreceptors	6 pA/Hz	*
J_GABA(LHA)-to-Ox_	C. strength of GABA on Ox neurons	118 pA/Hz	*
J_5-HT-to-Ox_	C. strength of 5-HT neurons on Ox neurons	6 pA/Hz	*
J_GABA(LHA)-to-GABA(LHA)_	C. strength of GABAergic neurons in LHA	5 pA/Hz	J_GABA(LHA)-on-GABA(LHA)_ ∼ J_GABA(DRN)-on-GABA(DRN)_
J_Ox-to-GABA(LHA)_	C. strength of Ox on GABAergic neurons in LHA	∼0	Very weak connection, [4]
J_5-HT-to-GABA(LHA)_	C. strength of 5-HT on LHA’s GABAergic neurons	6 pA/Hz	J_5-HT(DRN)-on-GABA(LHA)_ ∼J_5-HT(DRN)-on-Ox(LHA)_
I_Background-5-HT_	Background current amplitude in 5-HT neurons	231.47 pA	**
I_Background-Ox_	Background current amplitude in Ox neurons	617.6 pA	**
I_Background-GABA(DRN)_	Background current amplitude in DRN’s GABAergic neurons	246 pA	**
I_Background-GABA(LHA)_	Background current amplitude in LHA’s GABAergic neurons	20.9 pA	**

*Values of *J_syn,j,i_* are calculated from the current *I_j,i_* and the associated firing rate activity *f_i_* and also by using the above defined constraints.

**Parameter values are chosen such that the baseline activities and basic electrophysiological properties of the neurons are similar to those in experiments [4], [27], [35], [66], [71], [72], [73].

Thus the free parameters in the model are: *J_GABA(DRN)-to-GABA(DRN)_*, *J_GABA(LHA)-to-GABA(LHA)_* connection strengths of the GABAergic self-inhibition in DRN and LHA neuronal groups; *J_5-HT-to-GABA(LHA)_* connection strengths of the effect of 5-HT on GABAergic neurons in LHA; *τ_ 5-HT-on-GABA(LHA)_* time constants of the 5-HT effects on GABAergic neurons in LHA; *τ_Ox-on-GABA(LHA)_* time constant of the effect of Ox on GABAergic neurons in LHA; and afferent background currents to the neuronal groups, I_Background-5-HT_, I_Background-Ox_, I_Background-GABA(DRN)_, and I_Background-GABA(LHA)_.

In addition we make the following further constraints on the values of *J’s* and*τ’s*:

- Time constant of 5-HT effect on LHA’s GABAergic neurons is equivalent to that of 5-HT on LHA’s Ox neurons, i.e. *τ_5-HT-on-GABA(LHA)_∼τ_5-HT-on-Ox_*
- Time constants of Ox (Ox_2_) effect on GABAergic neurons in LHA is equivalent to the time constant of self-excitation of Ox (Ox_2_) autoreceptors in LHA, i.e. *τ_Ox-on-GABA(LHA)_∼ τ_Ox-auto_*
- Connection strengths of 5-HT on GABAergic neurons (LHA) are equivalent to the connection strengths of 5-HT on Ox neurons in LHA, i.e. *J_5-HT-to-GABA(LHA)_∼J_5-HT-to-Ox_*
- Connection strength of Ox on GABAergic neurons (LHA) is equivalent to the connection strength of the Ox autoreceptors in LHA, i.e. *J_Ox-to-GABA(LHA)_∼J_Ox-auto_*
- Connection strengths of GABAergic (GABA_A_) self-inhibition in DRN or LHA is equivalent, i.e. *J_GABA(LHA)-to-GABA(LHA)_*∼*J_GABA(DRN)-to-GABA(DRN)_*.

Further details of the model parameter values, justifications, and their related references are summarized in Table 1.

#### Simulations and analysis

We use XPPAUT [74] for neural circuit dynamics simulations and stability analysis. The Runge-Kutta 2 numerical integration algorithm with a time step of 10 ms is used. Smaller time steps do not affect our results.

## Results

### 5-HT_3A_R, 5-HT_1B_R, 5-HT_2A_R and 5-HT_2C_R Receptors on Ox Neurons in the LHA

**Figure 1:**
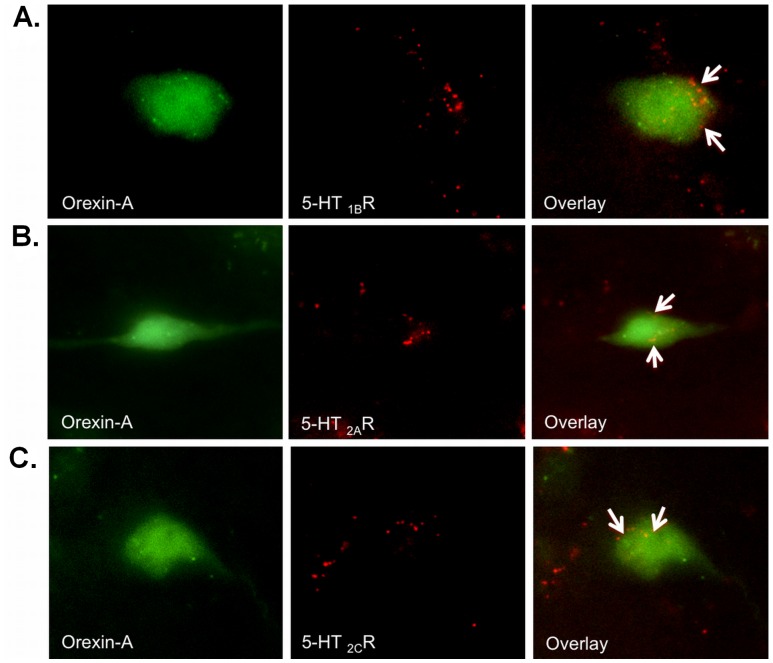
Single-label immunofluorescence analysis showing Ox neurons expressing additional 5HT receptor subtypes. (A) 5- HT_1B_R, (B) 5-HT_2A_R and (C) 5-HT_2C_R in the LHA of EGFP transgenic mice expressing GFP (green) exclusively in the Ox neurons. Immunoreactivity for 5-HT receptors (Alexa 546, red), 100× magnification.

**Figure 2:**
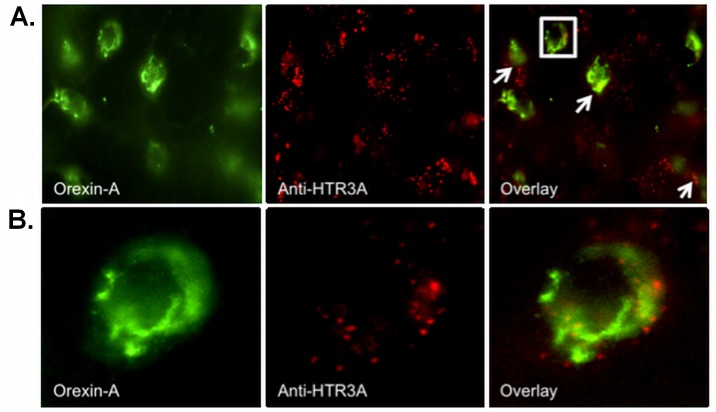
Double-label immunofluorescence analysis showing 5-HT_3A_R (anti-HTR3A) immunoreactivity of Ox (Orexin-A) neurons in the LHA. Confocal microscopy: Chromogens were Alexa 488 (green) and Alexa 546 (red), 100× magnification. (A) Arrows indicate the presence of 5-HT_3A_R on Ox neurons in the LHA. (B) Enlarged image of the selected square in A.

**Figure 3:**
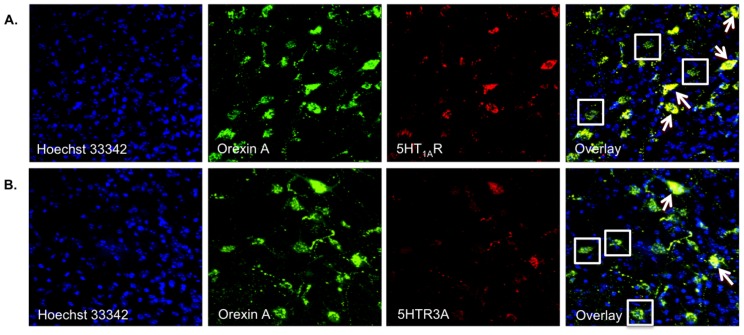
Representative z-stack_max_ (30 µm thickness) confocal images. Colocalization of Orexin A with (A) 5HT_1A_R, (B) 5HT_3A_R, 63× magnification. White arrows indicate orexin neuron expressing the respective 5HT receptor and white boxes indicate orexin neuron not expressing the receptor.

Muraki et al. (2004) have shown the presence of 5-HT_1A_ receptors on Ox neurons in the LHA. To elucidate the presence of additional 5-HT receptor subtypes on Ox neurons, we performed single IF staining for 5-HT_1B_R, 5-HT_2A_R and 5-HT_2C_R on Ox-EGFP transgenic mice brains (Figure 1). Co-localization of the receptor and the Ox neuron immunoreactivity (Figure 1A–C) can be observed in the respective overlay images. Using double IF labeling, we found fast ligand-based 5-HT_3A_ receptors on Ox neurons in the LHA of wild type C57BL/6 mice (Figure 2). This suggests that the DRN-to-LHA connection may transmit signals fast. Figure 3 shows representative z-stack_max_ (30 µm thickness) confocal images showing colocalization of Orexin A with 5HT_1A_R (Figure 3A) and 5HT_3A_R (Figure 3B), respectively.

**Figure 4:**
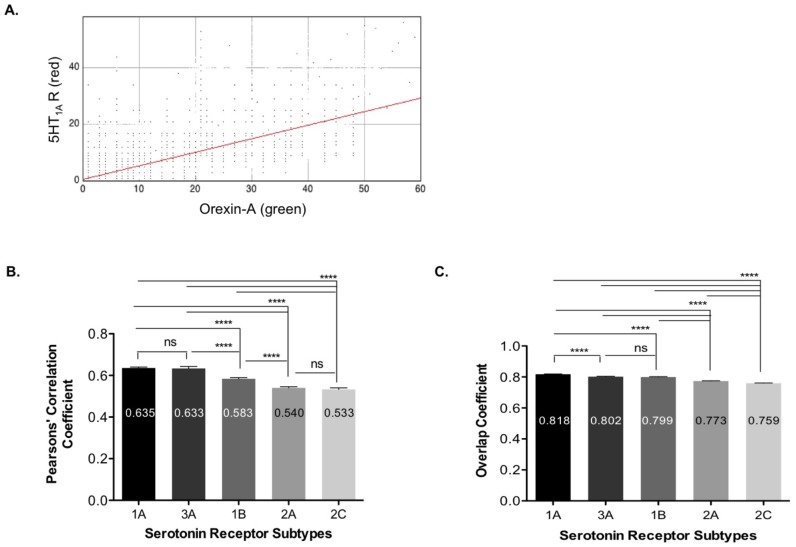
Quantitative analysis of the degree of colocalization of serotonin receptor subtypes and Ox neurons in the LHA. (A) Representative Scatter plot (cytofluorogram) indicating colocalization of 5HT_1A_R and Orexin A, Pearson’s coefficient = 0.641, (B) Pearson’s correlation coefficient calculated for the estimation of degree of colocalization of serotonin reseptor subtypes (5HT_1A_R, 5HT_3A_R, 5HT_1B_R, 5HT_2A_R and 5HT_2C_R) and Ox neurons in the LHA, (C) Overlap coefficient by Manders calculated for the estimation of extent of overlap of serotonin receptor subtypes (5HT_1A_R, 5HT_3A_R, 5HT_1B_R, 5HT_2A_R and 5HT_2C_R) and Ox neurons in the LHA. Data was analysed by two tailed unpaired t-test (Prism 5 software). Level of significance ****(p<0.0001).

As we demonstrated the additional 5HT receptor subtypes on the Ox neurons in LHA for the first time, the next obvious step was to quantify the double-labeled Ox neurons for each 5HT receptor subtype. Using the JACoP plug-in tool, we quantified the degree of co-localization by calculating Pearson’s correlation coefficient (Figure 4B) and overlap coefficient by Manders (Figure 4C). It is important to note, that the two coefficients, namely Pearson’s Correlation Coefficient and Overlap Coefficient, showed similar pattern of changes while revealing the different aspects of the colocalization process, proving the applicability of the calculations to investigate the degree of colocalization of serotonin receptor subtypes and orexin A (Ox neurons).

### 5-HT_3A_R and 5-HT_1A_R Receptors on Ox as well as GABAergic Interneurons in the LHA

**Figure 5:**
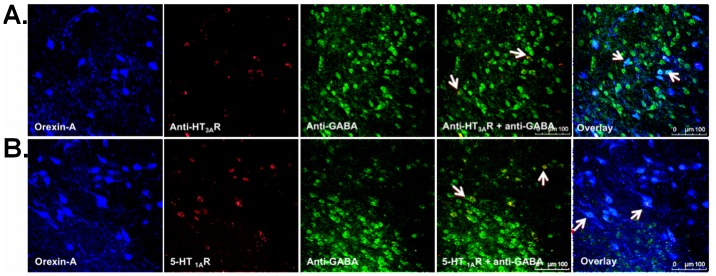
Representative photomicrographs of triple-label IF staining in LHA for Ox (Orexin-A) and GABAergic (anti-GABA) neurons. (A) 5-HT_3A_R, overlay image indicates an overlay of Orexin-A, 5-HT_3A_R and anti-GABA, (B) 5-HT_1A_R, overlay image indicates an overlay of Orexin-A, 5-HT_1A_R and anti-GABA. Confocal microscopy: Chromogens were Alexa 488 (blue), Alexa 546 (red) and CF633 (green), 40× magnification.

To examine the serotonergic long-range connections from DRN in the midbrain to the Ox and GABAergic neurons in the LHA, triple-label IF staining was performed. We searched for 5-HT_3A_R (Figure 5A) and 5-HT_1A_R (Figure 5B) on the Ox and GABAergic neurons and found that these 5-HT receptors may be present on Ox as well as local GABAergic neurons. This may suggest both direct and indirect (via LHA’s GABAergic neurons) 5-HT influence on the Ox neurons.

### OX_1_R, OX_2_R and NMDAR1 Receptors on Ox Neurons but not on GABAergic Neurons in the LHA

**Figure 6:**
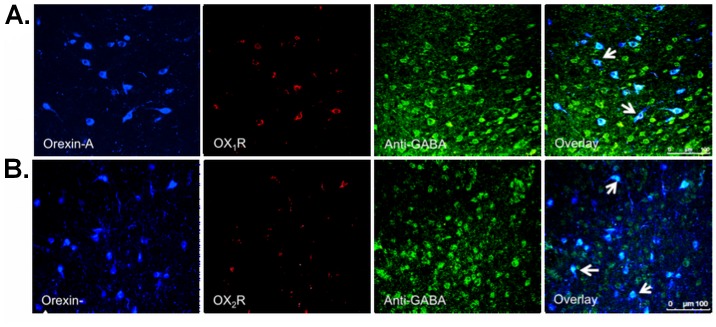
Representative photomicrographs of triple-label IF staining for Ox and GABAergic neurons in LHA. (A) OX_1_R, overlay image indicates an overlay of Orexin-A, OX_1_R and anti-GABA, (B) OX_2_R, overlay image indicates an overlay of Orexin-A, OX_2_R and anti-GABA. Arrows in A and B overlay images indicate presence of OX_1_R and OX_2_R on Ox neurons, respectively. Confocal microscopy: Chromogens were Alexa 488 (blue), Alexa 546 (red) and CF633 (green), 40×.

To identify the role of Ox neurons in regulating the activity of local GABAergic neurons, we carried out a triple IF labeling in the LHA. To study this inter-relationship, we searched for OX_1_R and OX_2_R receptors on the Ox and GABAergic neurons. We identified OX_1_R (Figure 6A) and OX_2_R (Figure 6B) on the Ox neurons but found these to be absent on the GABAergic interneurons. This sheds light on the self-regulatory mechanism of Ox neurons via OX_1_R and OX_2_R auto-receptors. Previous studies have demonstrated the presence of inhibitory GABA_B_ receptors on the Ox neurons [33], [75], [76], [77] and there is evidence that GABA_A_ receptors are also present on Ox neurons (Backberg et al., 2004, Kokare et al., 2006). This could suggest a one-way relationship between GABAergic and Ox neurons wherein GABAergic neurons exerts an inhibitory effect on the Ox neurons under the partial DRN’s serotonergic control, consistent with Yamanaka et al. (2010).

**Figure 7:**
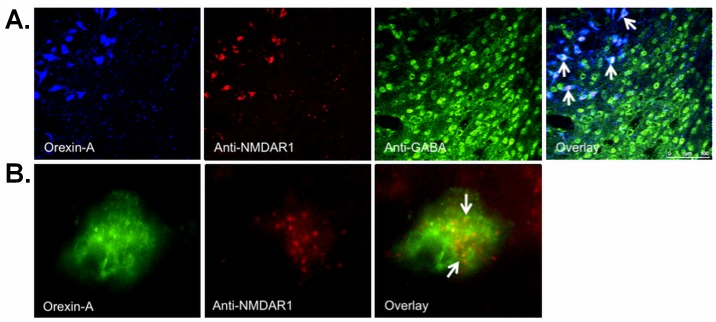
Representative photomicrographs of triple-label IF staining for NMDAR1 on Ox (Orexin-A) and GABAergic (anti-GABA) neurons. (A) NMDAR1 (anti-NMDAR1) in the LHA. (B) 100× magnification in A. Magenta colour in the overlay image in A and yellow/orange in the overlay image in B indicates the presence of NMDAR1 on Ox neurons. Confocal microscopy: Chromogenes were Alexa 488 (blue in A and green in B), Alexa 546 (red) and CF633 (green), 40×.

Ox neurons form a distinct group of a hypothalamic neuronal population that project to multiple brain regions and coordinate many physiological functions [9], [78]. Also, there is a convergence of signals that regulate the activity of Ox neurons by neurotransmitters, hormones, etc. Glutamate is an important neurotransmitter for Ox neurons and excitatory AMPA receptors have been shown to mediate the miniature EPSC in Ox neurons [79]. Also, NMDA receptors activate Ox neurons in the perifornical region of LHA [80]. To verify whether NMDA receptors are present on the overall Ox neurons and not confined solely to the perifornical area, we did triple-label IF staining. Here, we show the presence of N-methyl D-aspartate receptor 1 (NMDAR1) on the Ox neurons (Figure 7) and their absence on the GABAergic interneurons (Figure 7A, overlay image).

### Ox Axonal Projections Receive Glutamatergic and GABAergic Post-synaptic Inputs in the DRN

**Figure 8:**
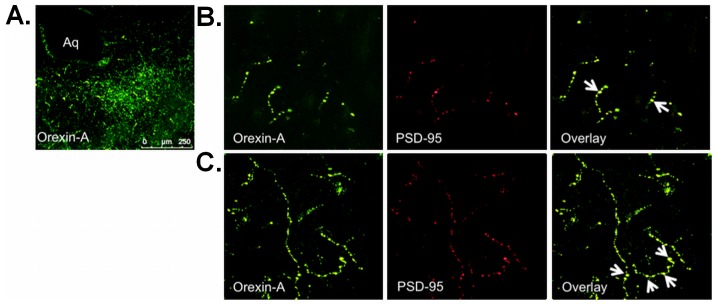
Ox axonal projections receive glutamatergic post-synaptic inputs in the DRN. (A) Ox (Orexin-A) axonal projections in the DRN, *Aq* indicates aqueduct, 20× magnification. (B) Double IF staining was done for Ox axons and PSD-95 (post-synaptic glutamatergic marker) in the DRN, 100×. Confocal microscopy: magnification 100×. (C) Z-stack_max_ image of 30 µm thickness selected from 45 µm section.

**Figure 9:**
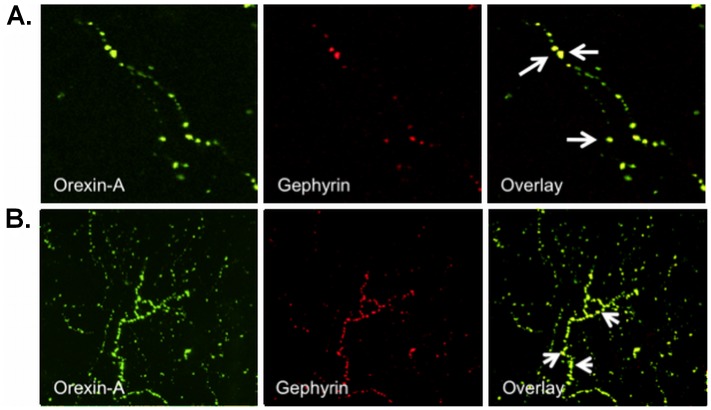
Ox axonal projections receive GABAergic post-synaptic inputs in the DRN. (A) Double IF staining was performed for Ox axons (Orexin-A) and Gephyrin (post-synaptic GABAergic marker) in the DRN. Confocal microscopy: magnification 100×. (B) Z-stack_max_ image of 30 µm thickness selected from 45 µm section.

To study the long-range connections of Ox neurons in the DRN, we performed IF labeling in the DRN region for PSD-95 (a marker for glutamatergic synapses) and gephyrin (a marker for GABAergic synapses) post-synaptic proteins [81]. Firstly, we carried out double-label IF staining for PSD-95 and Ox in the DRN and observed PSD-95 immunopositive Ox axonal terminals (Figure 8) indicating glutamatergic input to the Ox axonal projections. In another set of experiments, we examined GABAergic post-synaptic input to the Ox terminals as it has been shown that Ox fibres project to both 5-HT and GABAergic neurons in the DRN [31]. A double-label IF analysis for gephyrin and Ox in the DRN (Figure 9) showed gephyrin immunopositive Ox terminals, further indicating GABAergic input to the Ox projections. This result is in agreement with the findings of Liu et al. (2002) where they showed Ox axons in proximity to GABA/GABA-transporter immunoreactive neurons.

### Map of the DRN-LHA Circuit

**Figure 10:**
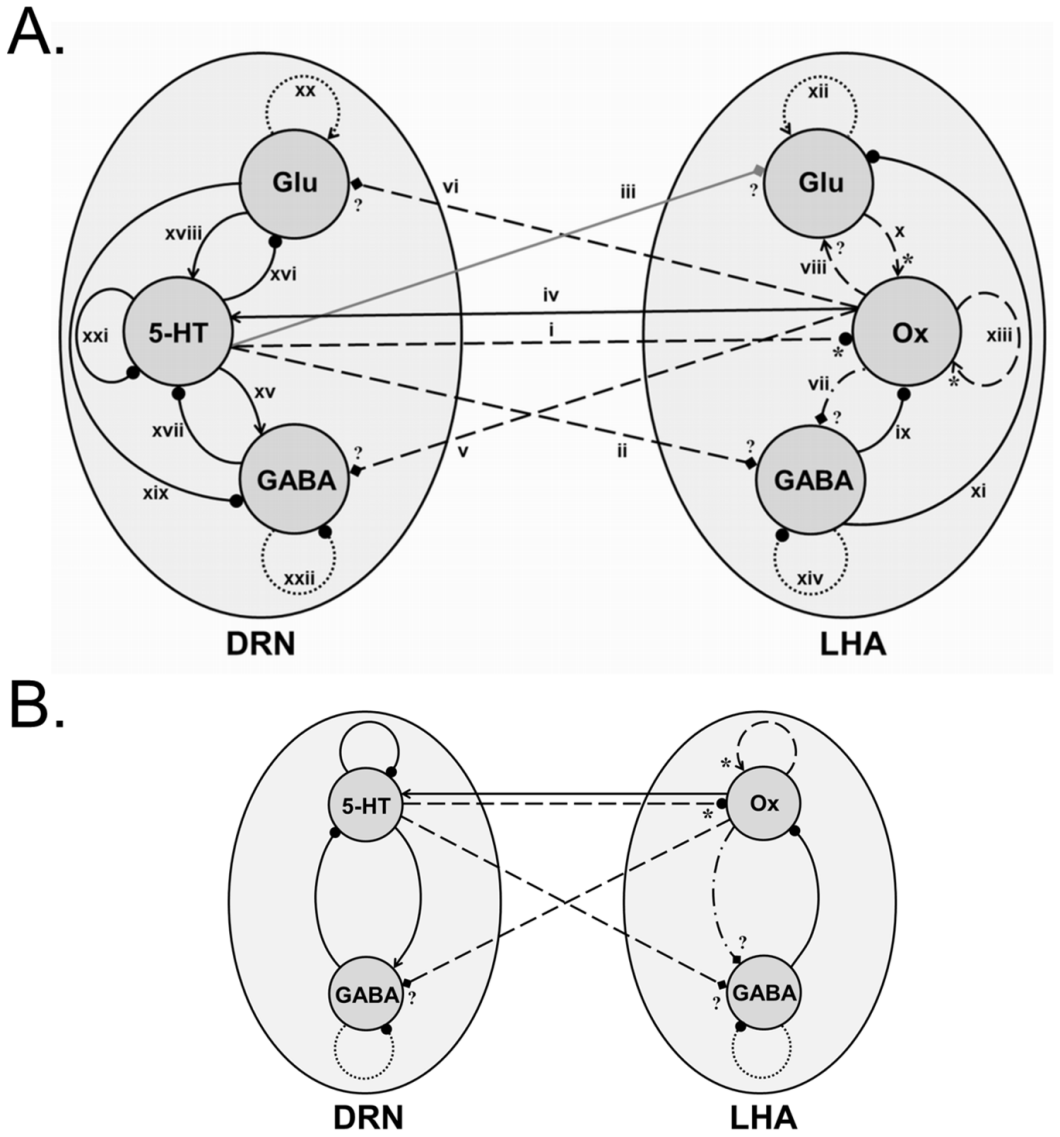
Neural circuit map of the LHA-DRN system. (A) Diagrammatic representation of the receptor mapping suggests a complex bi-directional relationship between DRN and LHA. Black circle/arrow represents effective inhibitory/excitatory connections between LHA and DRN, solid black line represents various receptor types derived from previous experimental studies, dashed black line shows the recent experimental findings, dashed arrows with * (asterisk) indicates that some of these receptors are known in the previous studies, dashed-dotted line signifies that receptors (in the target neurons) are not identified in this current study, dotted lines shows the hypothesized connection, diamond arrows indicate that connection types (excitatory/inhibitory) are not known, ? (question mark) means that receptors are not known and gray line signifies that the connection is not studied. All these receptor types are numbered on the figure and details are provided below. See text for more information. (B) Reduced LHA-DRN circuit used in the computational model. Label as in (A).

Based on the above results and in other previous studies (see below), a tentative map of the DRN and LHA is illustrated in Figure 10A. Connection (i) in the figure involves 5-HT_1A_R [26], 5-HT_1B_R, 5-HT_2A_R, 5-HT_2C_R, 5-HT_3A_R (found in our current study). Although connection (ii), found in this study, consists of 5-HT_1A_R and 5-HT_3A_R, the specific types of connection (e.g. effectively excitatory or inhibitory) and their strengths are not known. Connection (iii) and its receptor types are not known yet. Similarly, the receptor types for connection (iv) (Ox_1_R and Ox_2_R found in this study are consistent with previous findings [30], [32]), and connections (v) and (vi) (found in this study) are unknown. Connection (vii) receptors are not known and specifically Ox_1_R and Ox_2_R are not found in our study.

Other connections in the circuit based on other previous work: (viii) excitatory connection and receptor types are not known [82]; (ix) GABA_A/B_ [33], [75], [76], [77]; (x) AMPAR [79] and NMDAR1 (found in the current study); (xi) GABA_B_ [77]; (xiii) Ox_2_R [4], Ox_1_R and Ox_2_R (found in the current studies); (xv) 5-HT_1B/1D_ [34]; 5-HT_1A/2A/2C_ [69]; (xvi) 5-HT_7_ [34]; (xvii) GABA_A/B_ [34]; (xviii) AMPAR and NMDAR [34]; (xix) AMPAR and NMDAR [34]; (xxi) 5-HT_1A_R [83]. For the connections (xii), (xiv), (xx) and (xxii) (shown by black dotted circle/arrow in Figure 10A), we implicitly assume that non-principal neurons (GABAergic and glutamatergic neurons in LHA and DRN) are self-coupled.

### Neural Circuit Modelling and Analysis of the Direct and Indirect Interactions within the DRN-LHA System

The experimental findings in this study and in other previous work now provide sufficient information to build a computational model to investigate how the DRN-LHA neural architecture can influence the systems dynamics. We purposely simplified the implementation of the neural population units and connections in order to better illustrate the effects of network topology on dynamics (see Materials and Methods section). We also omit modelling the glutamatergic neurons in the DRN and LHA because there is evidence that glutamate has a weaker effect than GABA in the DRN [59]; and that indirect excitation from LHA’s glutamatergic neurons on Ox neurons may be represented implicitly by the Ox autoreceptors. This significantly reduces the number of model parameters and dynamical equations involved. In our model, we find that the various receptor subtypes will not affect the steady-state activities of the system. We shall henceforth not elaborate on the specific receptor subtype till we investigate the transient activation part of the results (section 3.6.3) when we study the influence of various timescales induced by the various receptors.

Next we investigate how the unknown model parameters and different timescales affect the system behaviour. Specifically, the focus is to understand the effects of: (i) 5-HT on LHA’s GABAergic neurons; (ii) local Ox and GABAergic interactions; and (iii) how connection timescales affect phasic 5-HT or Ox activations.

### Oscillatory DRN-LHA Behaviour if 5-HT Weakly Excites or Inhibits LHA’s GABAergic Neurons

**Figure 11:**
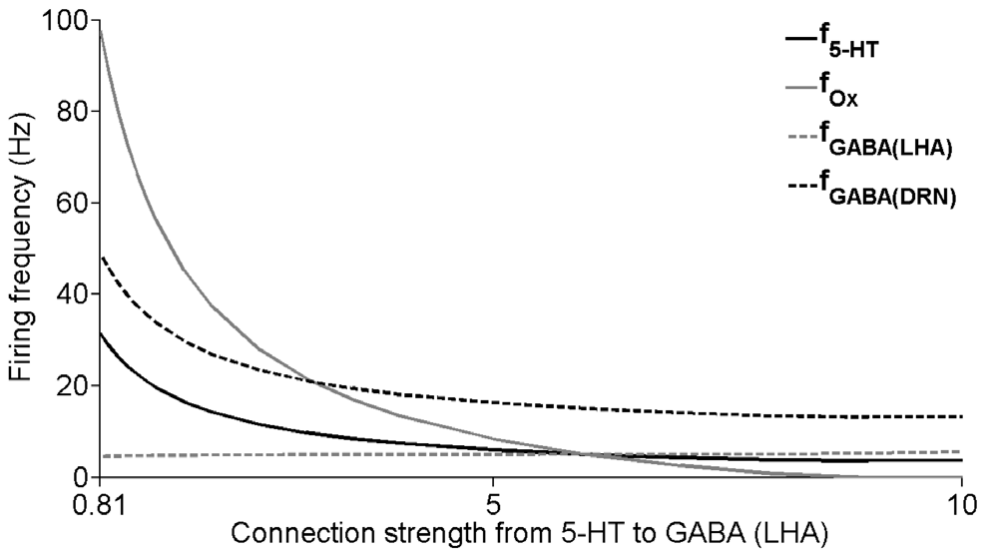
Neural circuit responses to change in connection strength from 5-HT to GABAergic neurons (LHA). Change in the steady-state values of the neural firing frequencies of the neuronal groups with varying connection strength factor J_5-HT-to-GABA(LHA)_ (in pA/Hz) from 5-HT neurons to the GABAergic neurons (LHA) for excitatory connection. Steady states are obtained after simulating for a sufficiently long time. f_5-HT_, f_Ox_, g_GABA(LHA)_, and g_GABA(DRN)_: population firing frequencies of 5-HT, Ox, LHA’s GABAergic and DRN’s GABAergic neurons, respectively.

It is not yet known whether the connection from 5-HT neurons to LHA’s GABAergic neurons (*J_5-HT-to-GABA(LHA)_*) is effectively excitatory or inhibitory. Here, we shall explore these possibilities. If this connection is excitatory, we find that as its connection strength *J_5-HT-to-GABA(LHA)_* is increased, the steady-state firing rate activities for most of the neural populations decrease (Figure 11). Note that LHA’s GABAergic neural population barely increases. This slight change is due to the self-inhibition within these GABAergic neurons. In contrast, the relatively larger changes for the other neural populations (especially when the connection strength is weaker) are due to the strong inhibitory projection from the LHA’s GABAergic neuron onto the Ox neurons (see Table 1), which subsequently affect the neurons in the DRN.

**Figure 12:**
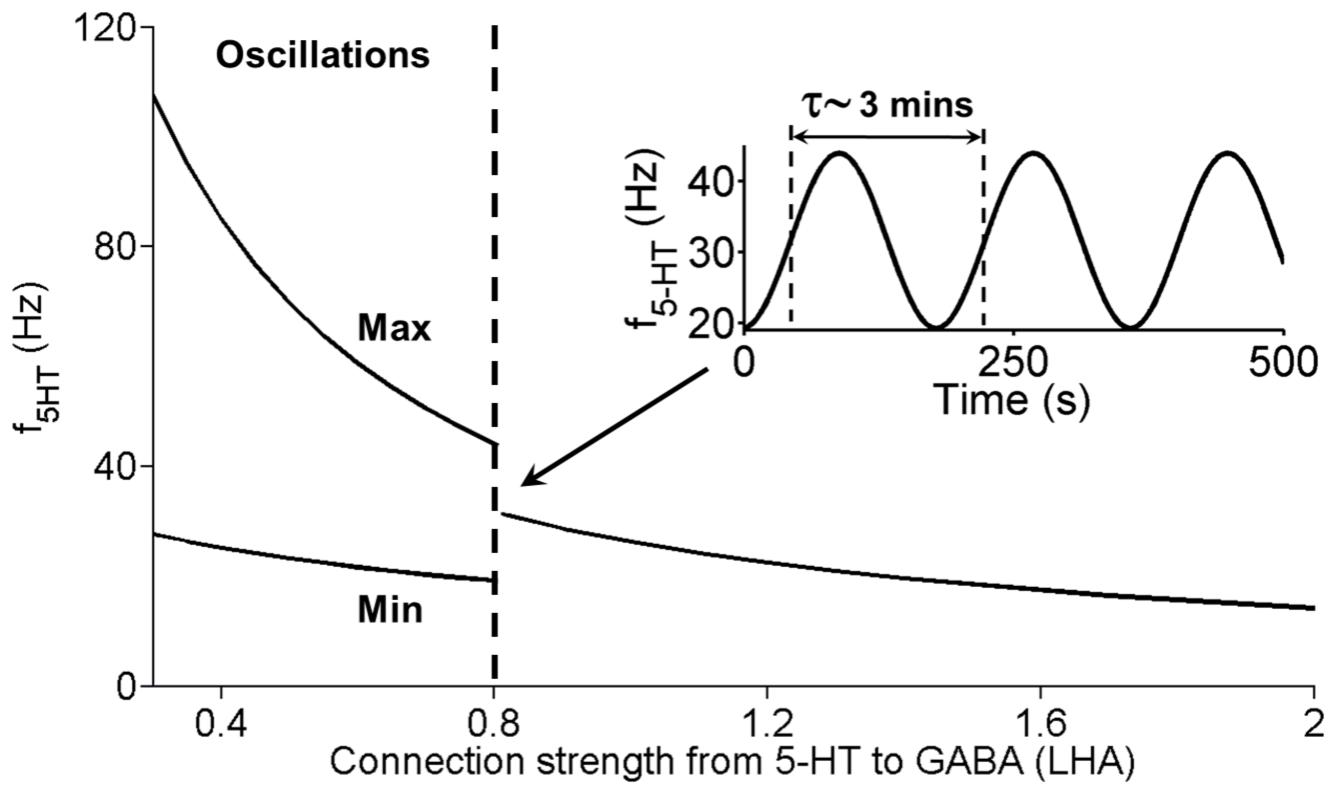
LHA-DRN system can exhibit oscillations with weak excitatory from 5-HT to GABAergic (LHA) neurons. Steady-state values of the firing rate activity of 5-HT neurons are plotted as a function of the connection strength J_5-HT-to-GABA(LHA)_ (in pA/Hz). Oscillatory region (left of dashed) is bounded by the values of J_5-HT-to-GABA(LHA)_ below 0.804 pA/Hz. Max (Min): maximum (minimum) firing rates during oscillation. Label as in Figure 11.

When the connection from 5-HT to LHA’s GABAergic neurons becomes very weak (*J_5-HT-to-GABA (LHA)_* close to 0), the DRN-LHA circuit will begin to exhibit slow oscillatory behaviour. Figure 12 shows such transition towards oscillatory behaviour for one sample neural population (the rest of the neural populations look similar). The oscillatory period can be as slow as a few minutes (Figure 12, inset). It can also be observed that as the connection strength decreases, the amplitude of oscillation (bounded by the top and bottom lines in grey region) increases. When this particular connection becomes inhibitory (*J_5-HT-to-GABA(LHA)_* <0), the oscillation amplitude can become very large (not shown). It is well-known that excitatory-inhibitory network can easily create oscillation [60], [61]. Similarly, the observed oscillation phenomenon can be explained by the interplay between strong inhibition and self-excitatory auto-regulation in the Ox neural population; the oscillation disappears when Ox autoreceptors are removed from the model e.g. when J_Ox-auto_ = 0 (not shown). As far as we know, there has yet to be any observable slow oscillation of Ox in the timescale of minutes. Hence, our model suggests that strong excitatory connection from 5-HT to LHA’s GABAergic neurons is more plausible, and we shall proceed with this assumption from here on.

### DRN-LHA System is Resilient to Changes in the Ox-to-GABAergic Neurons in the LHA

**Figure 13:**
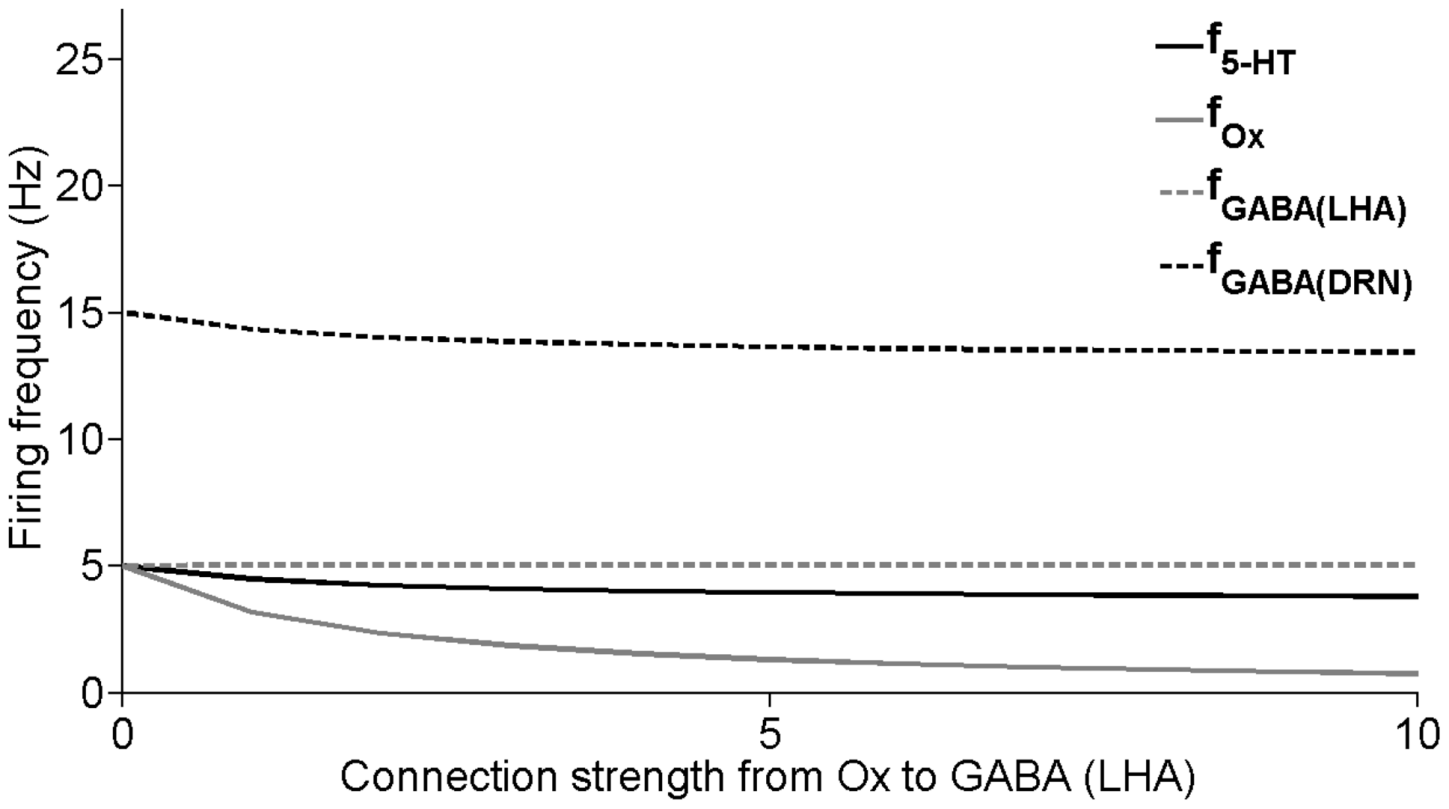
Neural circuit responses to change in connection strength from Ox to GABAergic neurons (LHA). Steady-state firing frequencies of the neural populations as functions of the connection strength (in pA/Hz). Label as in Figure 11.

One of our experimental findings in this study shows that the LHA’s GABAergic neurons do not have Ox receptors, consistent with indirect results from previous works [4]. Using our model, we check for the significance of such Ox receptors’ absence. In our model, we gradually increase the connection strength of Ox to LHA GABAergic neurons (*J_Ox-to-GABA(LHA)_*). This only marginally decreases the DRN-LHA steady-state activities (Figure 13). A similar explanation as that for the results in Figure 11 can be used to account for this phenomenon.

### Transient Ox and 5-HT Activities can be Affected by Different Receptor Timescales

Having studied the tonic activities (stable steady states) of the LHA-DRN system, we shall now proceed to investigate how transient or phasic activations of 5-HT or Ox can affect the system. The motivation for this is that it has been known that 5-HT and Ox can phasically activate in the presence of behaviourally relevant stimuli [84], [85], [86]. Furthermore, our current experimental finding suggests that 5-HT can influence LHA neurons over multiple timescales, through both the slow G-protein-coupled (non-5-HT_3A_) and fast ligand-gated (5-HT_3A_) receptors. To simulate this, the (more plausible) excitatory connection *J_5-HT-to-GABA(LHA)_* is considered, and a pulse stimulus current of the duration of 0.5 s with an amplitude of 150 pA is applied to either the 5-HT or Ox neural populations. To understand the individual role of the slow and fast timescales, and minimize any confounding effect, we simulate the two timescales separately.

**Figure 14:**
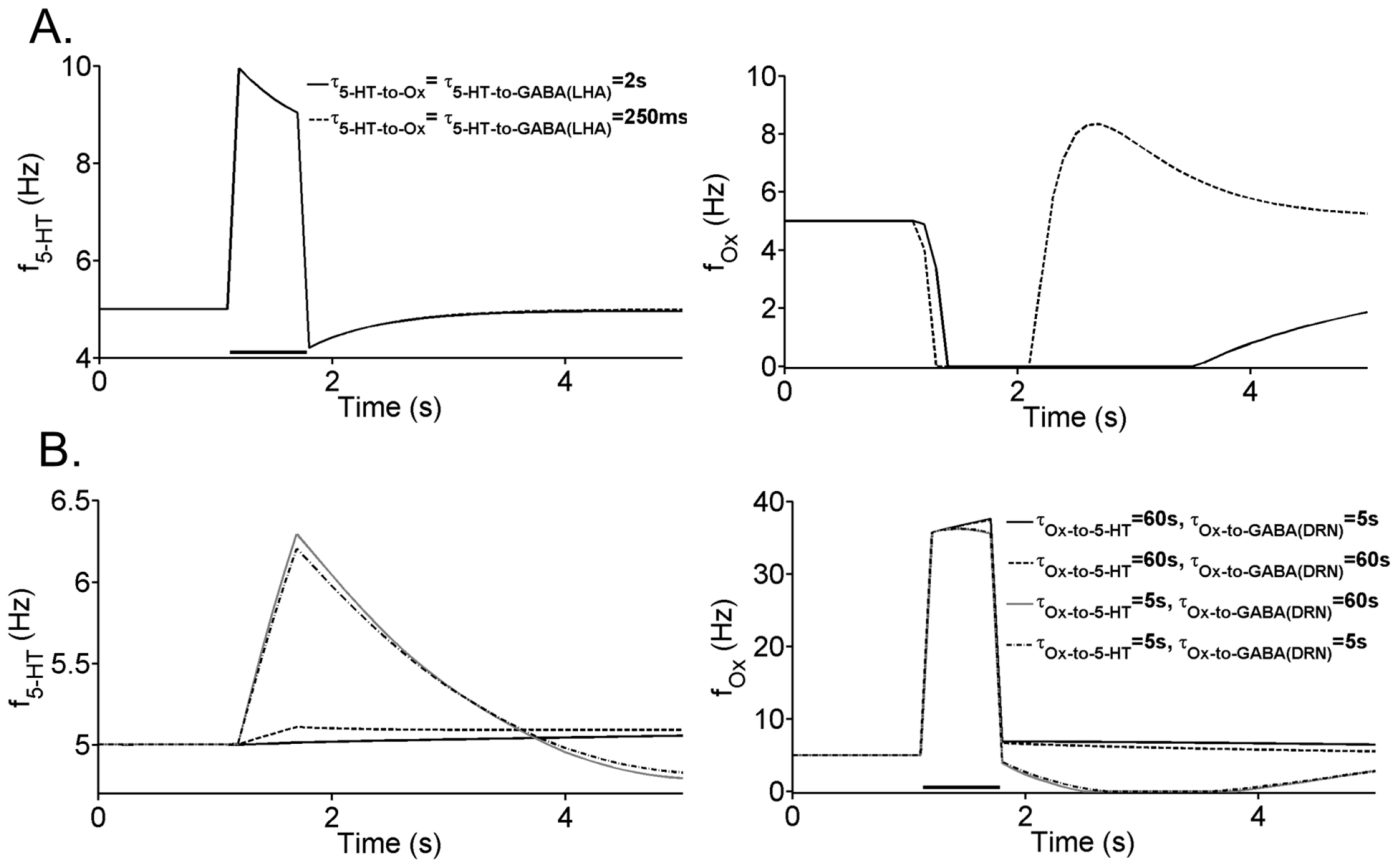
Transient activities of 5-HT and Ox neuronal groups under phasic stimulation. (A) Firing frequency of 5-HT neurons for different slow and fast 5-HT receptor timescales (left) and for the Ox neurons (right). (B) Firing frequency of 5-HT neurons for different slow and fast Ox receptor time scales (left) and for the Ox neurons (right). Solid horizontal black lines denote presence of stimulus with duration of 500 ms, and amplitude of 150 pA. Label as in Figure 11.

Figure 14A (left) shows that a 0.5 s stimulation of 5-HT neurons rapidly increase its activity, followed by a slower decay back towards baseline. The 5-HT activity decay is due to feedback inhibition from its autoreceptors and the local GABAergic neurons (hence the undershoot below baseline). The Ox activity responses are generally suppressed by the strong 5-HT-mediated inhibition (Figure 14A, right). When the 5-HT-to-Ox and 5-HT-to-GABA (LHA) connections are fast (mimicking 5-HT_3A_R), the rebound upon removal of stimulus (∼ after the 2 s mark in Figure 14A, right) is higher, suggesting the faster disinhibition than that for the slower connections. Varying these timescales do not affect the transient 5-HT activity in DRN (overlapping curves, Figure 14A, left).

When a similar stimulus is applied to the Ox neurons, the activity of the Ox neurons increases considerably throughout the stimulus duration (Figure 14B, right), which is due to the self-amplifying effect of Ox autoreceptors dominating over the local GABAergic inhibition (feedback inhibition is not strong due to the weak excitatory connection from Ox neurons to their local GABAergic neurons). These Ox neurons, affect 5-HT neurons either directly or indirectly (through the DRN’s GABAergic neurons). From our simulations, we find that when the Ox-to-5-HT connection acts on a fast timescale (5 s), they can transiently excite the 5-HT neurons (Figure 14B, left). If the direct timescale is much slower (e.g. 60 s), then 5-HT neurons can hardly be activated. Varying Ox-to-GABA(DRN) connection does not affect the system significantly. Note the post-stimulus undershoot due to recurrent inhibition, suppressing the Ox and 5-HT transiently below baseline. Taken together, when Ox-to-5-HT acts on a faster timescale, the phasic influence of Ox on 5-HT activity is much larger.

## Discussion

We have mapped out the direct and indirect connections between and within the DRN and LHA brain regions using IF staining to identify the receptors and trace long-range connections. To consider indirect connections, non-principal neurons have to be involved. In our study, the non-principal neurons are the inhibitory GABAergic and excitatory glutamatergic neurons, although there exist other neuronal types (e.g. neurons containing neuropeptide Y, etc) [87].

We have confirmed a previously identified 5-HT_1A_ receptor on Ox neurons, and have also identified multiple major 5-HT receptors in the LHA. They include, on Ox neurons, 5-HT_1B_R, 5-HT_2A_R, and 5-HT_2C_R and also fast ligand-gated 5-HT_3A_R. It is interesting to note that Muraki et al. (2004) has shown that the 5-HT_1A_ receptor antagonist WAY-100635 can completely block the 5-HT hyperpolarizing effect on Ox neurons. It could perhaps be that the 5-HT_1A_ receptors have the highest affinity as compared to the other 5-HT receptor subtypes found in this study. Further experimental work would help to clarify this issue. In addition, we found both fast and slow 5-HT receptors (5-HT_3A_R and 5-HT_1A_R) on LHA’s GABAergic neurons. Hence, these 5-HT (especially 1A) receptors could directly and indirectly influence Ox neurons, and further studies would be required to compare their relative affinities, and hence relative influences on Ox neurons. Within the LHA, we have also found the presence of OX_1_R, OX_2_R and NMDAR1 receptors on Ox neurons but not on GABAergic neurons. For LHA-to-DRN projections, we have identified Ox neurons receiving glutamatergic and GABAergic post-synaptic inputs in the DRN. The results from our experimental work and from previous work are summarized in Figure 10A. Our results also suggest that LHA’s GABAergic neurons could be isolated from direct (excitatory) afferent influences from local glutamatergic and Ox neurons, but could be influenced directly by 5-HT neurons, or through a unidirectional closed loop that involves the Ox and 5-HT neurons (Figure 10A).

From Figure 10B, the local LHA architecture looks similar in some respects to the well-studied cortical column architecture, with the Ox neurons acting as pyramidal neurons, with excitatory feedback among themselves [88]. However, our present work and in a previous study [4] have shown Ox neurons to have very weak influence on their local GABAergic neurons. This differs from the stereotypical excitatory-inhibitory feedback loop in a cortical column model. The DRN also looks similar to that of a cortical column if we include glutamatergic neurons (or glutamate-containing 5-HT neurons) to provide excitatory feedback. But excitation in the DRN is known to be weaker than GABA-mediated inhibition [59]. To provide a systemic understanding of the identified DRN-LHA architecture, we incorporated some of our current findings and previous published data into a computational model, which is an extension of our previous model [58].

We purposely simplified the neural unit and neuronal interaction implementations to focus primarily on this unique neural architecture of the DRN-LHA system. As a first step, glutamatergic neurons were justifiably omitted in our model simulations and analyses. We have included various constraints to the model, specifically on the relative values of the input-output slope, total input currents, and time constants and relative strengths of the connections. More importantly, we intentionally constructed a model to demonstrate the complex consequences of the neural circuit architecture we have established from our current experimental study.

We first use our model to explore the consequences of 5-HT’s effect on LHA’s GABAergic neurons. We found that the system becomes oscillatory when the connection strength is weak or inhibitory (Figure 12). This oscillatory behaviour has so far not been observed in experiments. Thus, based on these results, we hypothesize that this connection is excitatory. It would be interesting to test the strength of this connection using 5-HT_3A_R agonists/antagonists on LHA’s GABAergic neurons. This hypothesis would also imply that DRN may send inhibition to Ox neurons both directly and indirectly (through the GABAergic neurons), i.e. no balanced projections. This is in contrast with the long-range projection of Ox to the DRN, which excites both 5-HT neurons and GABAergic neurons in the DRN (Figure 10B).

Another interesting experimental finding in our work is the indication of an absence of Ox receptors on LHA’s GABAergic neurons. Our model simulations show that this particular connection does not affect the coupled DRN-LHA system significantly (Figure 13). This means that Ox receptor on LHA’s GABAergic neurons will have little influence on the circuit’s dynamics. It is interesting to speculate that the absence of these Ox receptors could be due to a lack of significant functional roles at the circuit level.

From our experimental work, we have identified both slow 5-HT G-protein-coupled and fast ligand-based receptors on both Ox and GABAergic neurons in the LHA. Our model attempted to mimic these different 5-HT receptor mediated timescales separately to investigate how the DRN-LHA circuit as a whole can be affected. We found that the 5-HT timescales do not change the tonic (steady-state) activities of the system, but can greatly affect the transient activations (Figure 14). In general, a faster transient 5-HT influence on Ox neurons does not affect the suppression much but can result in a faster disinhibition of Ox neurons. This could mean that the faster 5-HT_3A_R could be useful for quickly resetting the Ox neurons back to baseline after phasic 5-HT activation. More interestingly, a faster transient Ox influence can excite phasically 5-HT activity while slower timescale does not.

In summary, we have established aspects of the neurobiological circuitry function between the levels of 5-HT and Ox through direct and indirect pathways between the DRN and LHA. This work could have important implications in clinical neuroscience and neuropsychopharmacology as this DRN-LHA loop has been interpreted in two ways. It has been hypothesized that lower levels of 5-HT (common in depression) provide weaker inhibition to Ox neurons. Taking into account the effects of the circadian regulation of Ox and its influence on other neurotransmitters or neuromodulators, the Ox level may increase by this change in 5-HT. This increase in Ox levels may then generate a state similar to insomnia and other mood alterations. Similarly, in the reverse direction, it has been argued that lower levels of 5-HT are due to either a weaker excitatory connection from the Ox neurons (LHA) or because of lower levels of Ox (LHA), a situation commonly seen in hypersomnia or in narcolepsy [3], [89]. To explore the potential effects of Ox knock-out mice on 5-HT activity, our model can simulate such an effect by removing all the Ox effects in the circuit, and we observed that 5-HT neurons can still fire at ∼3.5 Hz (not shown). Furthermore, drug studies often do not consider integrating multiple targeted and non-targeted but connected brain areas. For example, in the DRN-LHA circuit considered in this study, an administration of 5-HT_1A_R agonist can directly affect not only 5-HT autoreceptors, but also all the 5-HT_1A_R in the DRN’s GABAergic neurons, and LHA’s Ox and GABAergic neurons. Other brain regions without 5-HT or Ox receptors, but connected to the affected DRN and LHA, will also be indirectly affected. Thus, the overall effect is complex, and this could be one important reason underlying serious side effects of various neuropharmacological drugs. A promising approach to gain a holistic understanding of such complex neurobiological systems is to perform more intensive computational modelling, simulations and analyses.

## References

[1] Saper CB, Scammell TE, Lu J (2005) Hypothalamic regulation of sleep and circadian rhythms. Nature 437: 1257–1263. 16251950 10.1038/nature04284

[2] Palasz A, Lapray D, Peyron C, Rojczyk-Golebiewska E, Skowronek R, et al.. (2013) Dual orexin receptor antagonists - promising agents in the treatment of sleep disorders. Int J Neuropsychopharmacol: 1–12. 10.1017/S1461145713000552 23702225

[3] Mignot E (2001) A commentary on the neurobiology of the hypocretin/orexin system. Neuropsychopharmacology 25: S5–13. 11682267 10.1016/S0893-133X(01)00316-5

[4] Yamanaka A, Tabuchi S, Tsunematsu T, Fukazawa Y, Tominaga M (2010) Orexin directly excites orexin neurons through orexin 2 receptor. J Neurosci 30: 12642–12652. 20861370 10.1523/JNEUROSCI.2120-10.2010 PMC6633594

[5] Feng P, Vurbic D, Wu Z, Hu Y, Strohl KP (2008) Changes in brain orexin levels in a rat model of depression induced by neonatal administration of clomipramine. J Psychopharmacol 22: 784–791. 18753273 10.1177/0269881106082899 PMC3580265

[6] Borgland SL, Labouebe G (2010) Orexin/hypocretin in psychiatric disorders: present state of knowledge and future potential. Neuropsychopharmacology 35: 353–354. 20010719 10.1038/npp.2009.119 PMC3055428

[7] Brundin L, Bjorkqvist M, Petersen A, Traskman-Bendz L (2007) Reduced orexin levels in the cerebrospinal fluid of suicidal patients with major depressive disorder. Eur Neuropsychopharmacol 17: 573–579. 17346943 10.1016/j.euroneuro.2007.01.005

[8] Salomon RM, Ripley B, Kennedy JS, Johnson B, Schmidt D, et al (2003) Diurnal variation of cerebrospinal fluid hypocretin-1 (Orexin-A) levels in control and depressed subjects. Biol Psychiatry 54: 96–104. 12873798 10.1016/s0006-3223(02)01740-7

[9] Lopez M, Tena-Sempere M, Dieguez C (2010) Cross-talk between orexins (hypocretins) and the neuroendocrine axes (hypothalamic-pituitary axes). Front Neuroendocrinol 31: 113–127. 19654017 10.1016/j.yfrne.2009.07.001

[10] Sakurai T, Mieda M, Tsujino N (2010) The orexin system: roles in sleep/wake regulation. Ann N Y Acad Sci 1200: 149–161. 20633143 10.1111/j.1749-6632.2010.05513.x

[11] de Lecea L, Kilduff TS, Peyron C, Gao X, Foye PE, et al (1998) The hypocretins: hypothalamus-specific peptides with neuroexcitatory activity. Proc Natl Acad Sci U S A 95: 322–327. 9419374 10.1073/pnas.95.1.322 PMC18213

[12] Sakurai T, Amemiya A, Ishii M, Matsuzaki I, Chemelli RM, et al (1998) Orexins and orexin receptors: a family of hypothalamic neuropeptides and G protein-coupled receptors that regulate feeding behavior. Cell 92: 573–585. 9491897 10.1016/s0092-8674(00)80949-6

[13] Urbanska A, Sokolowska P, Woldan-Tambor A, Bieganska K, Brix B, et al (2012) Orexins/hypocretins acting at Gi protein-coupled OX 2 receptors inhibit cyclic AMP synthesis in the primary neuronal cultures. J Mol Neurosci 46: 10–17. 21547533 10.1007/s12031-011-9526-2 PMC3260434

[14] Sakurai T (2005) Reverse pharmacology of orexin: from an orphan GPCR to integrative physiology. Regul Pept 126: 3–10. 15620407 10.1016/j.regpep.2004.08.006

[15] Xu TR, Ward RJ, Pediani JD, Milligan G (2012) Intramolecular fluorescence resonance energy transfer (FRET) sensors of the orexin OX1 and OX2 receptors identify slow kinetics of agonist activation. J Biol Chem. 10.1074/jbc.M111.334300 PMC3340242 22389503

[16] Scammell TE, Winrow CJ (2011) Orexin receptors: pharmacology and therapeutic opportunities. Annu Rev Pharmacol Toxicol 51: 243–266. 21034217 10.1146/annurev-pharmtox-010510-100528 PMC3058259

[17] Lanni C, Govoni S, Lucchelli A, Boselli C (2009) Depression and antidepressants: molecular and cellular aspects. Cell Mol Life Sci 66: 2985–3008. 19521663 10.1007/s00018-009-0055-x PMC11115917

[18] Adrien J (2002) Neurobiological bases for the relation between sleep and depression. Sleep Med Rev 6: 341–351. 12531125

[19] Wisor JP, Wurts SW, Hall FS, Lesch KP, Murphy DL, et al (2003) Altered rapid eye movement sleep timing in serotonin transporter knockout mice. Neuroreport 14: 233–238. 12598736 10.1097/00001756-200302100-00015

[20] Catena-Dell'osso M, Marazziti D, Rotella F, Bellantuono C (2012) Emerging targets for the pharmacological treatment of depression: focus on melatonergic system. Curr Med Chem 19: 428–437. 22335516 10.2174/092986712803414277

[21] Grace KP, Liu H, Horner RL (2012) 5-HT1A Receptor-Responsive Pedunculopontine Tegmental Neurons Suppress REM Sleep and Respiratory Motor Activity. J Neurosci 32: 1622–1633. 22302804 10.1523/JNEUROSCI.5700-10.2012 PMC6703359

[22] de Carvalho TB, Suman M, Molina FD, Piatto VB, Maniglia JV (2012) Relationship of obstructive sleep apnea syndrome with the 5-HT2A receptor gene in Brazilian patients. Sleep Breath. 10.1007/s11325-012-0645-y 22281949

[23] Artigas F (2013) Developments in the field of antidepressants, where do we go now? Eur Neuropsychopharmacol. 10.1016/j.euroneuro.2013.04.013 23706576

[24] Artigas F (2013) Serotonin receptors involved in antidepressant effects. Pharmacol Ther 137: 119–131. 23022360 10.1016/j.pharmthera.2012.09.006

[25] Li Y, van den Pol AN (2005) Direct and indirect inhibition by catecholamines of hypocretin/orexin neurons. J Neurosci 25: 173–183. 15634779 10.1523/JNEUROSCI.4015-04.2005 PMC6725201

[26] Muraki Y, Yamanaka A, Tsujino N, Kilduff TS, Goto K, et al (2004) Serotonergic regulation of the orexin/hypocretin neurons through the 5-HT1A receptor. J Neurosci 24: 7159–7166. 15306649 10.1523/JNEUROSCI.1027-04.2004 PMC6729168

[27] Yamanaka A, Muraki Y, Tsujino N, Goto K, Sakurai T (2003) Regulation of orexin neurons by the monoaminergic and cholinergic systems. Biochem Biophys Res Commun 303: 120–129. 12646175 10.1016/s0006-291x(03)00299-7

[28] Cooper MA, McIntyre KE, Huhman KL (2008) Activation of 5-HT1A autoreceptors in the dorsal raphe nucleus reduces the behavioral consequences of social defeat. Psychoneuroendocrinology 33: 1236–1247. 18692968 10.1016/j.psyneuen.2008.06.009 PMC2572256

[29] Brown RE, Sergeeva O, Eriksson KS, Haas HL (2001) Orexin A excites serotonergic neurons in the dorsal raphe nucleus of the rat. Neuropharmacology 40: 457–459. 11166339 10.1016/s0028-3908(00)00178-7

[30] Brown RE, Sergeeva OA, Eriksson KS, Haas HL (2002) Convergent excitation of dorsal raphe serotonin neurons by multiple arousal systems (orexin/hypocretin, histamine and noradrenaline). J Neurosci 22: 8850–8859. 12388591 10.1523/JNEUROSCI.22-20-08850.2002 PMC6757703

[31] Liu RJ, van den Pol AN, Aghajanian GK (2002) Hypocretins (orexins) regulate serotonin neurons in the dorsal raphe nucleus by excitatory direct and inhibitory indirect actions. J Neurosci 22: 9453–9464. 12417670 10.1523/JNEUROSCI.22-21-09453.2002 PMC6758063

[32] Soffin EM, Gill CH, Brough SJ, Jerman JC, Davies CH (2004) Pharmacological characterisation of the orexin receptor subtype mediating postsynaptic excitation in the rat dorsal raphe nucleus. Neuropharmacology 46: 1168–1176. 15111023 10.1016/j.neuropharm.2004.02.014

[33] Matsuki T, Nomiyama M, Takahira H, Hirashima N, Kunita S, et al (2009) Selective loss of GABA(B) receptors in orexin-producing neurons results in disrupted sleep/wakefulness architecture. Proc Natl Acad Sci U S A 106: 4459–4464. 19246384 10.1073/pnas.0811126106 PMC2657380

[34] Harsing LG Jr, Prauda I, Barkoczy J, Matyus P, Juranyi Z (2004) A 5-HT7 heteroreceptor-mediated inhibition of [3H]serotonin release in raphe nuclei slices of the rat: evidence for a serotonergic-glutamatergic interaction. Neurochem Res 29: 1487–1497. 15260125 10.1023/b:nere.0000029560.14262.39

[35] Lee HS, Park SH, Song WC, Waterhouse BD (2005) Retrograde study of hypocretin-1 (orexin-A) projections to subdivisions of the dorsal raphe nucleus in the rat. Brain Res 1059: 35–45. 16153616 10.1016/j.brainres.2005.08.016

[36] Kumar S, Szymusiak R, Bashir T, Rai S, McGinty D, et al (2007) Effects of serotonin on perifornical-lateral hypothalamic area neurons in rat. Eur J Neurosci 25: 201–212. 17241281 10.1111/j.1460-9568.2006.05268.x

[37] Postnova S, Voigt K, Braun HA (2009) A mathematical model of homeostatic regulation of sleep-wake cycles by hypocretin/orexin. J Biol Rhythms 24: 523–535. 19926811 10.1177/0748730409346655

[38] Williams KS, Behn CG (2011) Dynamic interactions between orexin and dynorphin may delay onset of functional orexin effects: a modeling study. J Biol Rhythms 26: 171–181. 21454297 10.1177/0748730410395471

[39] Patriarca M, Postnova S, Braun HA, Hernandez-Garcia E, Toral R (2012) Diversity and noise effects in a model of homeostatic regulation of the sleep-wake cycle. PLoS Comput Biol 8: e1002650. 22927806 10.1371/journal.pcbi.1002650 PMC3426568

[40] Carter ME, Brill J, Bonnavion P, Huguenard JR, Huerta R, et al (2012) Mechanism for Hypocretin-mediated sleep-to-wake transitions. Proc Natl Acad Sci U S A 109: E2635–2644. 22955882 10.1073/pnas.1202526109 PMC3465396

[41] Diniz Behn CG, Kopell N, Brown EN, Mochizuki T, Scammell TE (2008) Delayed orexin signaling consolidates wakefulness and sleep: physiology and modeling. J Neurophysiol 99: 3090–3103. 18417630 10.1152/jn.01243.2007 PMC3065358

[42] Rempe MJ, Best J, Terman D (2010) A mathematical model of the sleep/wake cycle. J Math Biol 60: 615–644. 19557415 10.1007/s00285-009-0276-5

[43] Kumar R, Bose A, Mallick BN (2012) A mathematical model towards understanding the mechanism of neuronal regulation of wake-NREMS-REMS states. PLoS One 7: e42059. 22905114 10.1371/journal.pone.0042059 PMC3414531

[44] Omenetti A, Yang L, Gainetdinov RR, Guy CD, Choi SS, et al (2011) Paracrine modulation of cholangiocyte serotonin synthesis orchestrates biliary remodeling in adults. Am J Physiol Gastrointest Liver Physiol 300: G303–315. 21071507 10.1152/ajpgi.00368.2010 PMC3043647

[45] Arenkiel BR, Peca J, Davison IG, Feliciano C, Deisseroth K, et al (2007) In vivo light-induced activation of neural circuitry in transgenic mice expressing channelrhodopsin-2. Neuron 54: 205–218. 17442243 10.1016/j.neuron.2007.03.005 PMC3634585

[46] Hu Z, Rudd JA, Fang M (2012) Development of the human corpus striatum and the presence of nNOS and 5-HT2A receptors. Anat Rec (Hoboken) 295: 127–131. 22095614 10.1002/ar.21497

[47] Johansson S, Povlsen GK, Edvinsson L (2012) Expressional changes in cerebrovascular receptors after experimental transient forebrain ischemia. PLoS One 7: e41852. 22848635 10.1371/journal.pone.0041852 PMC3407123

[48] Wai MS, Lorke DE, Kwong WH, Zhang L, Yew DT (2011) Profiles of serotonin receptors in the developing human thalamus. Psychiatry Res 185: 238–242. 20538346 10.1016/j.psychres.2010.05.003

[49] Yeung LY, Kung HF, Yew DT (2010) Localization of 5-HT1A and 5-HT2A positive cells in the brainstems of control age-matched and Alzheimer individuals. Age (Dordr) 32: 483–495. 20508993 10.1007/s11357-010-9152-x PMC2980600

[50] Ren LQ, Wienecke J, Chen M, Moller M, Hultborn H, et al.. (2013) The time course of serotonin 2C receptor expression after spinal transection of rats: an immunohistochemical study. Neuroscience. 10.1016/j.neuroscience.2012.12.063 23337537

[51] Weber M, Schmitt A, Wischmeyer E, Doring F (2008) Excitability of pontine startle processing neurones is regulated by the two-pore-domain K+ channel TASK-3 coupled to 5-HT2C receptors. Eur J Neurosci 28: 931–940. 18691333 10.1111/j.1460-9568.2008.06400.x

[52] Rivera HM, Santollo J, Nikonova LV, Eckel LA (2012) Estradiol increases the anorexia associated with increased 5-HT(2C) receptor activation in ovariectomized rats. Physiol Behav 105: 188–194. 21889523 10.1016/j.physbeh.2011.08.018 PMC3225592

[53] Zinchuk V, Grossenbacher-Zinchuk O (2009) Recent advances in quantitative colocalization analysis: focus on neuroscience. Prog Histochem Cytochem 44: 125–172. 19822255 10.1016/j.proghi.2009.03.001

[54] Zinchuk V, Zinchuk O, Okada T (2007) Quantitative colocalization analysis of multicolor confocal immunofluorescence microscopy images: pushing pixels to explore biological phenomena. Acta Histochem Cytochem 40: 101–111. 17898874 10.1267/ahc.07002 PMC1993886

[55] Bolte S, Cordelieres FP (2006) A guided tour into subcellular colocalization analysis in light microscopy. J Microsc 224: 213–232. 17210054 10.1111/j.1365-2818.2006.01706.x

[56] Adler J, Parmryd I (2010) Quantifying colocalization by correlation: the Pearson correlation coefficient is superior to the Mander's overlap coefficient. Cytometry A 77: 733–742. 20653013 10.1002/cyto.a.20896

[57] Costes SV, Daelemans D, Cho EH, Dobbin Z, Pavlakis G, et al (2004) Automatic and quantitative measurement of protein-protein colocalization in live cells. Biophys J 86: 3993–4003. 15189895 10.1529/biophysj.103.038422 PMC1304300

[58] Joshi A, Wong-Lin K, McGinnity TM, Prasad G (2011) A mathematical model to explore the interdependence between the serotonin and orexin/hypocretin systems. Conf Proc IEEE Eng Med Biol Soc 2011: 7270–7273. 22256017 10.1109/IEMBS.2011.6091837

[59] Tao R, Auerbach SB (2003) Influence of inhibitory and excitatory inputs on serotonin efflux differs in the dorsal and median raphe nuclei. Brain Res 961: 109–120. 12535783 10.1016/s0006-8993(02)03851-9

[60] Wilson HR, Cowan JD (1972) Excitatory and inhibitory interactions in localized populations of model neurons. Biophys J 12: 1–24. 4332108 10.1016/S0006-3495(72)86068-5 PMC1484078

[61] Dayan P, Abbott L (2011) Theoretical Neuroscience: Computational and Mathematical Modeling of Neural Systems. The MIT Press.

[62] Shriki O, Hansel D, Sompolinsky H (2003) Rate models for conductance-based cortical neuronal networks. Neural Comput 15: 1809–1841. 14511514 10.1162/08997660360675053

[63] Wong KF, Wang XJ (2006) A recurrent network mechanism of time integration in perceptual decisions. J Neurosci 26: 1314–1328. 16436619 10.1523/JNEUROSCI.3733-05.2006 PMC6674568

[64] Crawford LK, Craige CP, Beck SG (2010) Increased intrinsic excitability of lateral wing serotonin neurons of the dorsal raphe: a mechanism for selective activation in stress circuits. J Neurophysiol 103: 2652–2663. 20237311 10.1152/jn.01132.2009 PMC2867584

[65] Karnani MM, Szabo G, Erdelyi F, Burdakov D (2013) Lateral hypothalamic GAD65 neurons are spontaneously firing and distinct from orexin- and melanin-concentrating hormone neurons. J Physiol 591: 933–953. 23184514 10.1113/jphysiol.2012.243493 PMC3591707

[66] Kirby LG, Pernar L, Valentino RJ, Beck SG (2003) Distinguishing characteristics of serotonin and non-serotonin-containing cells in the dorsal raphe nucleus: electrophysiological and immunohistochemical studies. Neuroscience 116: 669–683. 12573710 10.1016/s0306-4522(02)00584-5 PMC2832757

[67] Katayama J, Yakushiji T, Akaike N (1997) Characterization of the K+ current mediated by 5-HT1A receptor in the acutely dissociated rat dorsal raphe neurons. Brain Res 745: 283–292. 9037420 10.1016/s0006-8993(96)01141-9

[68] Williams JT, Colmers WF, Pan ZZ (1988) Voltage- and ligand-activated inwardly rectifying currents in dorsal raphe neurons in vitro. J Neurosci 8: 3499–3506. 3171686 10.1523/JNEUROSCI.08-09-03499.1988 PMC6569458

[69] Liu R, Jolas T, Aghajanian G (2000) Serotonin 5-HT(2) receptors activate local GABA inhibitory inputs to serotonergic neurons of the dorsal raphe nucleus. Brain Res 873: 34–45. 10915808 10.1016/s0006-8993(00)02468-9

[70] Lee MG, Hassani OK, Jones BE (2005) Discharge of identified orexin/hypocretin neurons across the sleep-waking cycle. J Neurosci 25: 6716–6720. 16014733 10.1523/JNEUROSCI.1887-05.2005 PMC6725432

[71] Mileykovskiy BY, Kiyashchenko LI, Siegel JM (2005) Behavioral correlates of activity in identified hypocretin/orexin neurons. Neuron 46: 787–798. 15924864 10.1016/j.neuron.2005.04.035 PMC8281334

[72] Takahashi K, Lin JS, Sakai K (2008) Neuronal activity of orexin and non-orexin waking-active neurons during wake-sleep states in the mouse. Neuroscience 153: 860–870. 18424001 10.1016/j.neuroscience.2008.02.058

[73] Sakai K (2011) Sleep-waking discharge profiles of dorsal raphe nucleus neurons in mice. Neuroscience 197: 200–224. 21958868 10.1016/j.neuroscience.2011.09.024

[74] Ermentrout GB, Kopell N (1990) Oscillator death in systems of coupled neural oscillators. SIAM Journal on Applied Mathematics 50: 125–146.

[75] Xie X, Crowder TL, Yamanaka A, Morairty SR, Lewinter RD, et al (2006) GABA(B) receptor-mediated modulation of hypocretin/orexin neurones in mouse hypothalamus. J Physiol 574: 399–414. 16627567 10.1113/jphysiol.2006.108266 PMC1817779

[76] Backberg M, Ultenius C, Fritschy JM, Meister B (2004) Cellular localization of GABA receptor alpha subunit immunoreactivity in the rat hypothalamus: relationship with neurones containing orexigenic or anorexigenic peptides. J Neuroendocrinol 16: 589–604. 15214862 10.1111/j.1365-2826.2004.01207.x

[77] Kokare DM, Patole AM, Carta A, Chopde CT, Subhedar NK (2006) GABA(A) receptors mediate orexin-A induced stimulation of food intake. Neuropharmacology 50: 16–24. 16168444 10.1016/j.neuropharm.2005.07.019

[78] Peyron C, Tighe DK, van den Pol AN, de Lecea L, Heller HC, et al (1998) Neurons containing hypocretin (orexin) project to multiple neuronal systems. J Neurosci 18: 9996–10015. 9822755 10.1523/JNEUROSCI.18-23-09996.1998 PMC6793310

[79] Alberto CO, Hirasawa M (2010) AMPA receptor-mediated miniature EPSCs have heterogeneous time courses in orexin neurons. Biochem Biophys Res Commun 400: 707–712. 20816937 10.1016/j.bbrc.2010.08.132

[80] Doane DF, Lawson MA, Meade JR, Kotz CM, Beverly JL (2007) Orexin-induced feeding requires NMDA receptor activation in the perifornical region of the lateral hypothalamus. Am J Physiol Regul Integr Comp Physiol 293: R1022–1026. 17537834 10.1152/ajpregu.00282.2007

[81] Henny P, Jones BE (2006) Innervation of orexin/hypocretin neurons by GABAergic, glutamatergic or cholinergic basal forebrain terminals evidenced by immunostaining for presynaptic vesicular transporter and postsynaptic scaffolding proteins. J Comp Neurol 499: 645–661. 17029265 10.1002/cne.21131 PMC2426825

[82] Li Y, Gao XB, Sakurai T, van den Pol AN (2002) Hypocretin/Orexin excites hypocretin neurons via a local glutamate neuron-A potential mechanism for orchestrating the hypothalamic arousal system. Neuron 36: 1169–1181. 12495630 10.1016/s0896-6273(02)01132-7

[83] Verge D, Daval G, Patey A, Gozlan H, el Mestikawy S, et al (1985) Presynaptic 5-HT autoreceptors on serotonergic cell bodies and/or dendrites but not terminals are of the 5-HT1A subtype. Eur J Pharmacol 113: 463–464. 2931289 10.1016/0014-2999(85)90099-8

[84] Ranade SP, Mainen ZF (2009) Transient firing of dorsal raphe neurons encodes diverse and specific sensory, motor, and reward events. J Neurophysiol 102: 3026–3037. 19710375 10.1152/jn.00507.2009

[85] Bromberg-Martin ES, Hikosaka O, Nakamura K (2010) Coding of task reward value in the dorsal raphe nucleus. J Neurosci 30: 6262–6272. 20445052 10.1523/JNEUROSCI.0015-10.2010 PMC3467971

[86] Wong-Lin K, Joshi A, Prasad G, McGinnity TM (2012) Network properties of a computational model of the dorsal raphe nucleus. Neural Netw 32: 15–25. 22386598 10.1016/j.neunet.2012.02.009

[87] Diaz-Cabiale Z, Parrado C, Narvaez M, Puigcerver A, Millon C, et al (2011) Galanin receptor/Neuropeptide Y receptor interactions in the dorsal raphe nucleus of the rat. Neuropharmacology 61: 80–86. 21396946 10.1016/j.neuropharm.2011.03.002

[88] Mountcastle VB (1997) The columnar organization of the neocortex. Brain 120 (Pt 4): 701–722. 10.1093/brain/120.4.701 9153131

[89] Ebrahim IO, Sharief MK, de Lacy S, Semra YK, Howard RS, et al (2003) Hypocretin (orexin) deficiency in narcolepsy and primary hypersomnia. J Neurol Neurosurg Psychiatry 74: 127–130. 12486284 10.1136/jnnp.74.1.127 PMC1738182

[90] Morikawa H, Manzoni OJ, Crabbe JC, Williams JT (2000) Regulation of central synaptic transmission by 5-HT(1B) auto- and heteroreceptors. Mol Pharmacol 58: 1271–1278. 11093763 10.1124/mol.58.6.1271

[91] Ishibashi H, Kuwano K, Takahama K (2000) Inhibition of the 5-HT(1A) receptor-mediated inwardly rectifying K(+) current by dextromethorphan in rat dorsal raphe neurones. Neuropharmacology 39: 2302–2308. 10974313 10.1016/s0028-3908(00)00092-7

[92] Gocho Y, Sakai A, Yanagawa Y, Suzuki H, Saitow F (2013) Electrophysiological and pharmacological properties of GABAergic cells in the dorsal raphe nucleus. J Physiol Sci 63: 147–154. 23275149 10.1007/s12576-012-0250-7 PMC3579464

[93] Yamanaka A, Muraki Y, Ichiki K, Tsujino N, Kilduff TS, et al (2006) Orexin neurons are directly and indirectly regulated by catecholamines in a complex manner. J Neurophysiol 96: 284–298. 16611835 10.1152/jn.01361.2005

